# Cannabinoids in oncology: pharmacology, clinical applications, and translational perspectives

**DOI:** 10.3389/fphar.2026.1969657

**Published:** 2026-09-29

**Authors:** Emma Safran, Howard Safran, Stanley Kim

**Affiliations:** 1 University of New England College of Osteopathic Medicine, Portland, ME, United States; 2 Division of Hematology and Oncology, The Miriam Hospital, Warren Alpert Medical School of Brown University, Providence, RI, United States

**Keywords:** cannabinoid, cannabis, cbd, drug interaction, endocannabinoid system, oncology, supportive care, thc

## Abstract

Cannabis and cannabinoid products are increasingly used by patients with cancer for symptom management and supportive care. Although cannabis is widely used to alleviate cancer- and treatment-related symptoms, many patients also believe that it possesses direct anticancer activity. This review provides a comprehensive pharmacologic and clinical assessment of cannabinoids in oncology, integrating the endocannabinoid system, cannabinoid receptor signaling, pharmacokinetics, therapeutic applications, adverse effects, drug–drug interactions, and emerging antitumor research. The strongest clinical evidence supports selected cannabinoid preparations for refractory chemotherapy-induced nausea and vomiting, whereas evidence for cancer-related pain, appetite stimulation, sleep disturbance, anxiety, and opioid-sparing effects remains limited or inconsistent. Δ^9^-tetrahydrocannabinol (THC) and cannabidiol (CBD) differ substantially in receptor pharmacology, psychoactive effects, metabolism, and toxicity, with important implications for product selection, dosing, and interactions with anticancer and supportive-care medications. Preclinical studies demonstrate antiproliferative, proapoptotic, antiangiogenic, and immunomodulatory effects across multiple tumor models; however, cannabinoid signaling is highly context dependent and may also promote tumor progression in selected settings. Human evidence for direct anticancer activity remains sparse, consisting predominantly of small early-phase studies and observational data, with no established improvement in tumor response, progression-free survival, or overall survival. Particular caution is warranted regarding neuropsychiatric and cardiovascular toxicity, pharmacokinetic drug interactions, and the potential influence of cannabis on immune checkpoint inhibitor outcomes. Overall, cannabinoids have defined but limited roles in supportive oncology, while their direct antitumor potential remains investigational. Future studies should prioritize standardized formulations, pharmacologically informed dosing, rigorous randomized designs, clinically meaningful endpoints, and biomarker-based approaches to define therapeutic benefit and risk.

## 1 Background

Cannabis has been used medicinally for thousands of years to treat pain, gastrointestinal disorders, muscle spasms, and numerous other conditions. Historical records document its therapeutic use in ancient China, India, Egypt, and the Middle East for more than 4,000 years, making it one of the oldest continuously used herbal medicines (Russo, 2007; National Academies of Sciences, 2017). During the twentieth century, concerns regarding psychoactive effects, abuse potential, and psychiatric complications led to strict regulatory restrictions that substantially limited both clinical use and scientific investigation (National Academies of Sciences, 2017). Over the past two decades, evolving legislation and growing public interest have renewed the medical use of cannabis and cannabinoid-based products, particularly among patients with chronic illnesses, including cancer (National Academies of Sciences, 2017; Skórzewska and Gęca, 2024).

The discovery of the endocannabinoid system (ECS) transformed the understanding of cannabinoid biology. Identification of cannabinoid receptor type 1 (CB1) in 1988, followed by cloning of CB1, discovery of cannabinoid receptor type 2 (CB2), and identification of the endogenous ligand anandamide established the ECS as a previously unrecognized physiological signaling network (Devane et al., 1988; Matsuda et al., 1990; Munro et al., 1993; Devane et al., 1992). The ECS is now recognized as a highly conserved regulatory system involved in neurotransmission, pain perception, appetite, energy metabolism, immune regulation, inflammation, and cellular homeostasis. Dysregulation of the ECS has been implicated in numerous pathological conditions, including neurological disorders, inflammatory diseases, metabolic disorders, and cancer, making it an attractive therapeutic target (Di M et al., 2015; Zou and Kumar, 2018; Cristino et al., 2020).

Cannabis use among patients with cancer has increased substantially (Pergam et al., 2017; Braun et al., 2018). Surveys suggest that approximately 20%–40% of patients use cannabis during the course of their illness, primarily to alleviate pain, chemotherapy-induced nausea and vomiting, anorexia, cachexia, insomnia, anxiety, and other treatment-related symptoms (Pergam et al., 2017). Many patients also believe that cannabinoids possess direct anticancer activity or may enhance conventional therapies. However, these expectations are based predominantly on preclinical observations, and convincing clinical evidence supporting cannabinoids as anticancer therapy remains lacking (Skórzewska and Gęca, 2024; Pergam et al., 2017; Braun et al., 2018).

Experimental studies have demonstrated that Δ^9^-tetrahydrocannabinol (THC), cannabidiol (CBD), and other phytocannabinoids exert antiproliferative, proapoptotic, antiangiogenic, antimetastatic, and immunomodulatory effects in cell culture and animal models (Guzmán, 2003; Velasco et al., 2012). Proposed mechanisms include inhibition of tumor-cell proliferation, induction of apoptosis and autophagy, suppression of angiogenesis and epithelial–mesenchymal transition, modulation of oxidative stress, and remodeling of the tumor immune microenvironment (Guzmán, 2003; Velasco et al., 2012). These findings provide a compelling biological rationale for investigating cannabinoids in oncology but have not translated into consistent clinical benefit (Skórzewska and Gęca, 2024; Guzmán, 2003; Velasco et al., 2012; Whiting et al., 2015).

To date, randomized clinical trials have not demonstrated reproducible improvements in objective response, progression-free survival, or overall survival with cannabinoid therapy (Whiting et al., 2015; Braun et al., 2024). In contrast, cannabinoids have an established role in selected supportive-care settings, particularly for chemotherapy-induced nausea and vomiting refractory to guideline-directed antiemetic therapy (Braun et al., 2024; Herrstedt et al., 2024; National Comprehensive Cancer Network, 2026). Evidence supporting their use for cancer-related pain, anorexia, cachexia, anxiety, and sleep disturbance remains limited and heterogeneous (Whiting et al., 2015; Braun et al., 2024). Increasing cannabinoid use has also heightened concerns regarding neuropsychiatric and cardiovascular toxicity, product quality and labeling inconsistencies, clinically important drug interactions, and potential interactions with anticancer therapies (Braun et al., 2024; Huestis, 2007; Zendulka et al., 2016).

The rapidly expanding cannabinoid literature presents important challenges for oncologists. Despite increasing patient interest, education regarding medical cannabis remains limited, resulting in considerable variability in physician knowledge, confidence, and clinical practice (Braun et al., 2018; Braun et al., 2024). Consequently, clinicians are increasingly required to provide balanced, evidence-based guidance regarding the benefits, limitations, and risks of cannabinoid therapy while interpreting a rapidly evolving and methodologically heterogeneous literature.

In this review, we summarize cannabinoid pharmacology and endocannabinoid-system biology, critically evaluate evidence supporting supportive-care applications, review preclinical and clinical studies of potential anticancer activity across major malignancies, discuss adverse effects and clinically important drug interactions, summarize recommendations from contemporary oncology societies, and highlight key knowledge gaps and future research priorities.

A literature search was conducted using PubMed/MEDLINE and Google Scholar to identify relevant English-language publications on cannabis, cannabinoids, the endocannabinoid system, supportive oncology, anticancer activity, adverse effects, and drug–drug interactions. Search terms included combinations of cannabis, cannabinoids, tetrahydrocannabinol, THC, cannabidiol, CBD, endocannabinoid system, cancer, oncology, cancer pain, chemotherapy-induced nausea and vomiting, anticancer, antitumor, drug interactions, and individual tumor types. Publications from database inception through July 2026 were considered, with emphasis on systematic reviews, randomized and prospective clinical studies, clinical practice guidelines, and mechanistically informative preclinical studies. Additional relevant publications were identified from the reference lists of selected articles. Studies were selected on the basis of relevance to the scope of the review and their contribution to the clinical or translational questions addressed. This article is a narrative review and was not conducted according to a systematic-review protocol; study selection therefore involved author judgment.

## 2 Cannabis pharmacology

### 2.1 Phytocannabinoids and cannabinoid-based medicines

Cannabis refers to plants of the genus *Cannabis*, particularly *Cannabis sativa* L. (Figure 1), which have been cultivated for medicinal, recreational, and industrial purposes for thousands of years (Russo, 2007; National Academies of Sciences, 2017; Skórzewska and Gęca, 2024). Although the terms *cannabis* and *marijuana* are frequently used interchangeably, *cannabis* denotes the botanical plant, whereas *marijuana* generally refers to its dried flowers, leaves, and related preparations used for medical or recreational purposes (Russo, 2007; National Academies of Sciences, 2017). Extensive hybridization among commercially cultivated varieties has rendered the traditional distinctions among *Cannabis sativa*, *Cannabis indica*, and *Cannabis ruderalis* of limited taxonomic and pharmacological relevance. Instead, the biological properties of medicinal cannabis are determined primarily by its cannabinoid, terpene, and broader phytochemical composition (Skórzewska and Gęca, 2024; Rock and Parker, 2021; Andre et al., 2016; Hanuš et al., 2016).

**FIGURE 1:**
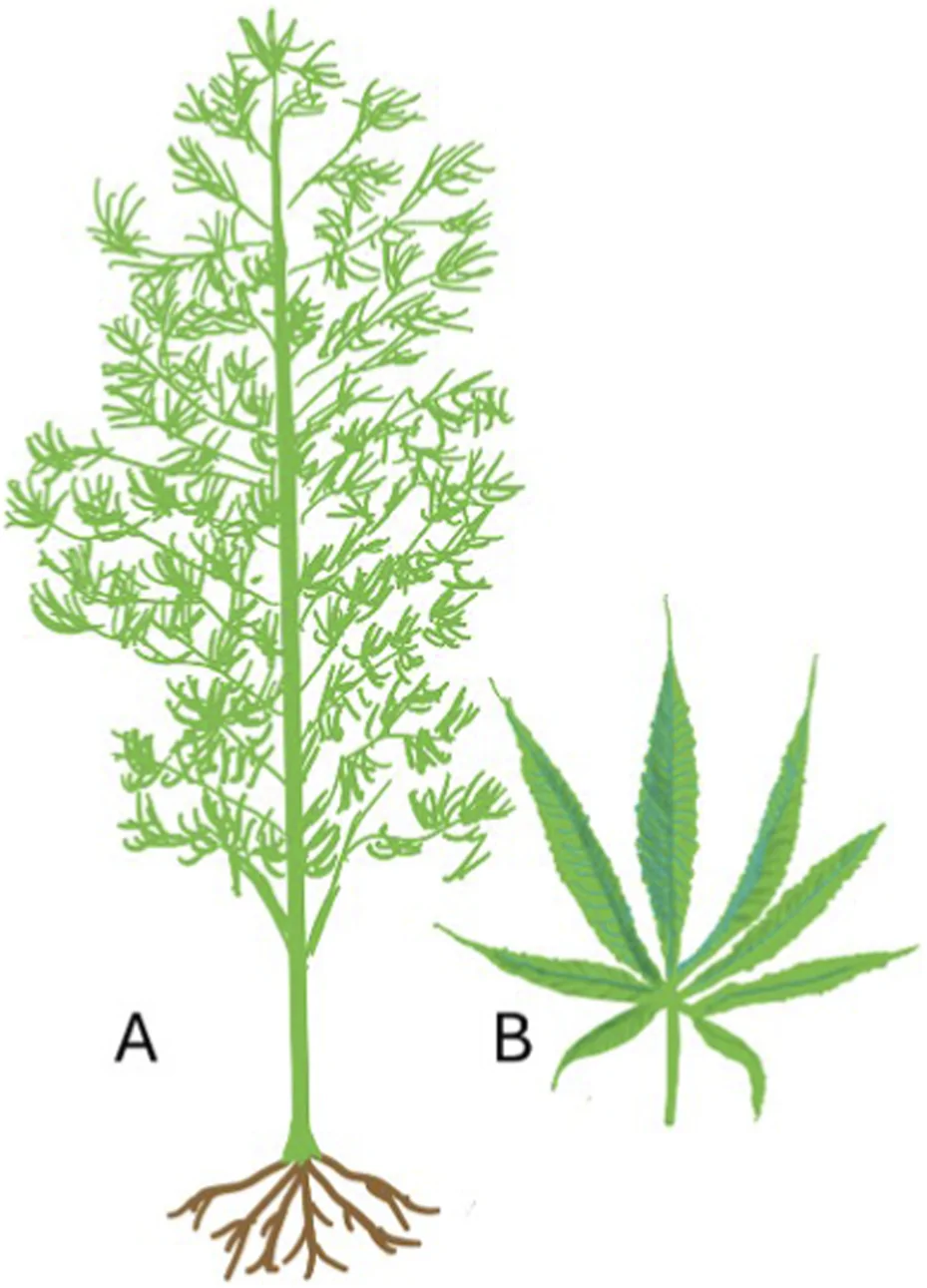
Cannabis sativa Plant and Leaf. **(A)** Whole Cannabis sativa plant. **(B)** Characteristic palmately compound leaf.

More than 500 chemical constituents have been identified in cannabis, including over 120 phytocannabinoids, together with terpenes, flavonoids, alkaloids, and numerous other bioactive compounds (Rock and Parker, 2021; Andre et al., 2016; Hanuš et al., 2016). THC and CBD are the principal phytocannabinoids responsible for most clinically relevant pharmacological effects. THC is a partial agonist of CB1 and CB2 receptors and contributes to analgesic, antiemetic, appetite-stimulating, and muscle-relaxant effects while also producing dose-dependent intoxication, cognitive impairment, anxiety, and psychomotor slowing (Howl et al., 2002; Mackie, 2008; Ibeas Bih et al., 2015; Morales et al., 2017; Velasco et al., 2016; Ladin et al., 2016; Dariš et al., 2019; Tomko et al., 2020). In contrast, CBD has relatively low affinity for CB1 and CB2 receptors and lacks the characteristic intoxicating effects of THC at therapeutic doses. It exhibits anticonvulsant, anti-inflammatory, anxiolytic, antioxidant, and immunomodulatory properties through multiple receptor-dependent and receptor-independent mechanisms (Huestis, 2007; Zendulka et al., 2016; Ibeas Bih et al., 2015; Morales et al., 2017; Velasco et al., 2016; Ladin et al., 2016).

The major phytocannabinoids, their molecular targets, pharmacological effects, and potential clinical applications are summarized in Table 1.

**TABLE 1:** Major phytocannabinoids, pharmacological properties, and potential clinical applications.

Cannabinoid	Psychoactive	Principal molecular targets	Major pharmacological effects	Potential clinical applications	References
Δ^9^-Tetrahydrocannabinol (THC)	Yes	CB1, CB2	Analgesic, antiemetic, appetite stimulant, muscle relaxant	Refractory CINV; appetite stimulation; investigational/individualized use for cancer-related symptoms	(Braun et al., 2024; Rock and Parker, 2021; Howl et al., 2002; Pertwee, 2008)
Cannabidiol (CBD)	Non-intoxicating	TRPV1, TRPV2, GPR55, PPARγ, 5-HT1A, adenosine pathways (minimal CB1/CB2 affinity)	Anti-inflammatory, anticonvulsant, anxiolytic, antioxidant, immunomodulatory; potential antitumor activity	Epilepsy; investigational supportive-oncology applications; investigational anticancer therapy	(Rock and Parker, 2021; Ibeas Bih et al., 2015; Morales et al., 2017; U.S. Food and Drug Administration, 2024b)
Cannabigerol (CBG)	No	Weak CB1/CB2 activity, TRP channels, α2-adrenergic receptors	Anti-inflammatory, antibacterial, neuroprotective; potential antiproliferative activity	Investigational	(Rock and Parker, 2021; Andre et al., 2016; Hanuš et al., 2016; Morales et al., 2017)
Cannabichromene (CBC)	No	TRPA1, TRPV1, TRPV3	Anti-inflammatory, analgesic; potential antiproliferative activity	Investigational	(Rock and Parker, 2021; Andre et al., 2016; Hanuš et al., 2016; Morales et al., 2017)
Cannabinol (CBN)	Mild	Weak CB1/CB2 activity	Mild sedative, analgesic, anti-inflammatory	Investigational	(Rock and Parker, 2021; Andre et al., 2016; Hanuš et al., 2016; Pertwee, 2008)
Tetrahydrocannabivarin (THCV)	Dose dependent*	CB1, CB2	Appetite modulation, metabolic effects; potential antitumor activity	Investigational	(Hanuš et al., 2016; Morales et al., 2017; Pertwee, 2008)

* THCV may exhibit different CB1 activity depending on dose and experimental conditions.

Abbreviations: CBD, cannabidiol; CBC, cannabichromene; CBG, cannabigerol; CBN, cannabinol; CB1, cannabinoid receptor type 1; CB2, cannabinoid receptor type 2; CINV, chemotherapy-induced nausea and vomiting; GPR55, G protein-coupled receptor 55; PPARγ, peroxisome proliferator-activated receptor-γ; TRP, transient receptor potential; THC, Δ^9^-tetrahydrocannabinol; THCV, tetrahydrocannabivarin.

Cannabis also contains numerous terpenes, including myrcene, limonene, β-caryophyllene, α-pinene, and linalool, which contribute to its characteristic aroma and may possess independent biological activity (Rock and Parker, 2021; Andre et al., 2016; Hanuš et al., 2016; Tomko et al., 2020). The proposed “entourage effect,” whereby cannabinoids and terpenes interact synergistically to enhance therapeutic efficacy or reduce adverse effects, remains biologically plausible but has not been confirmed in adequately powered randomized clinical trials (Rock and Parker, 2021; Andre et al., 2016; Hanuš et al., 2016; Tomko et al., 2020).

Industrial hemp is a category of *Cannabis sativa* cultivated primarily for fiber, textiles, food products, seed oil, and cannabinoid extraction. Under U.S. federal law, hemp contains no more than 0.3% Δ^9^-THC by dry weight. Although this concentration is generally insufficient to produce clinically significant intoxication, commercially available hemp-derived products exhibit considerable variability in cannabinoid content, purity, contaminants, and labeling accuracy (Skórzewska and Gęca, 2024; Whiting et al., 2015; Rock and Parker, 2021).

Botanical cannabis has not been approved by the U.S. Food and Drug Administration (FDA) for the treatment of cancer or any other medical condition. However, several cannabinoid-based pharmaceutical products have received regulatory approval. Dronabinol and nabilone are approved for chemotherapy-induced nausea and vomiting refractory to conventional antiemetic therapy, and dronabinol is additionally approved for AIDS-associated anorexia (U.S. Fo od and Drug Administration, 2017; U.S. Food and Drug Administration, 2024a; U.S. Food and Drug Administration, 2006). Epidiolex®, a highly purified plant-derived CBD formulation, is approved for seizures associated with Lennox–Gastaut syndrome, Dravet syndrome, and tuberous sclerosis complex (U.S. Food and Drug Administration, 2024b). Nabiximols, approximately 1:1 THC:CBD oromucosal spray, is approved in several countries—but not in the United States—for multiple sclerosis-related spasticity and has also been investigated for cancer pain and recurrent glioblastoma, although it is not an established anticancer therapy (Therapeutics Limited, 2026). The regulatory status, approved indications, and routes of administration of cannabinoid-based medications are summarized in Table 2.

**TABLE 2:** Approved cannabinoid medications.

Drug (Brand)	Active component	Approval status	Approved indications	Route of administration	References
Dronabinol (Marinol®, Syndros®)	Synthetic THC	FDA (U.S.)	Refractory chemotherapy-induced nausea and vomiting (CINV); AIDS-related anorexia	Oral capsule; oral solution	(U.S. Fo od and Drug Administration, 2017; U.S. Food and Drug Administration, 2024a)
Nabilone (Cesamet®)	Synthetic THC analogue	FDA (U.S.)	Refractory CINV	Oral capsule	(U.S. Food and Drug Administration, 2006)
Cannabidiol (Epidiolex®)	Purified plant-derived CBD	FDA (U.S.)	Lennox–Gastaut syndrome; Dravet syndrome; tuberous sclerosis complex	Oral solution	(U.S. Food and Drug Administration, 2024b)
Nabiximols (Sativex®)	THC:CBD (∼1:1 ratio)	Approved outside the U.S.; regulatory status varies by jurisdiction*	Multiple sclerosis–related spasticity	Oromucosal spray	(Therapeutics Limited, 2026)

* Regulatory approvals and indications vary by country and jurisdiction.

Abbreviations: CBD, cannabidiol; CINV, chemotherapy-induced nausea and vomiting; FDA, U.S. Food and Drug Administration; THC, Δ^9^-tetrahydrocannabinol.

### 2.2 The endocannabinoid system and cancer biology

The discovery of the endocannabinoid system (ECS) provided the biological foundation for understanding cannabinoid pharmacology and investigating the role of cannabinoids in cancer and other diseases (Devane et al., 1988; Matsuda et al., 1990; Munro et al., 1993; Devane et al., 1992; Di M et al., 2015; Zou and Kumar, 2018; Cristino et al., 2020). The ECS comprises cannabinoid receptors, endogenous ligands (endocannabinoids), and the enzymes responsible for their synthesis and degradation (Figure 2) (Di M et al., 2015; Zou and Kumar, 2018; Cristino et al., 2020).

**FIGURE 2:**
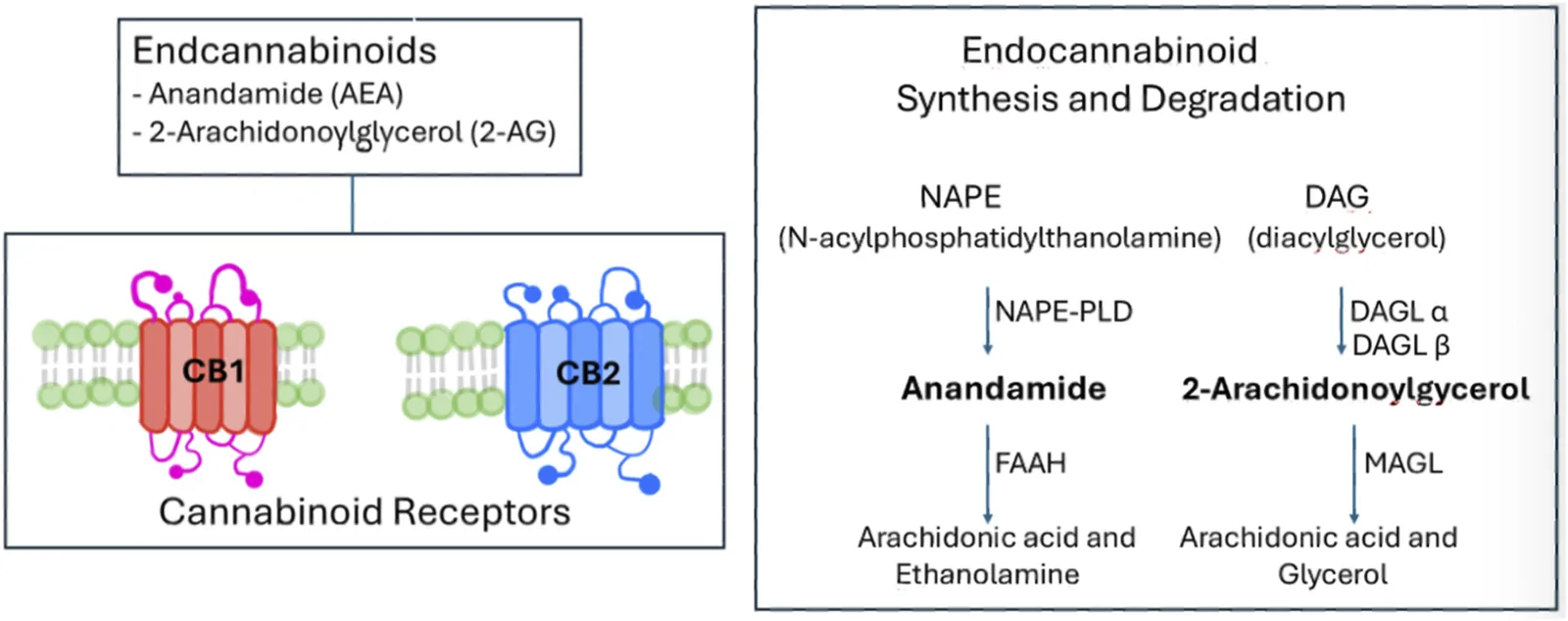
The endocannabinoid System. The endocannabinoid system comprises the endogenous cannabinoids AEA and 2-AG, cannabinoid receptors CB1 and CB2, and enzymes involved in endocannabinoid synthesis and degradation. AEA is synthesized principally from NAPE by NAPE-PLD and degraded primarily by FAAH. 2-AG is synthesized from DAG by DAGLα and DAGLβ and degraded primarily by MAGL. Abbreviations: AEA, anandamide; 2-AG, 2-arachidonoylglycerol; CB1, cannabinoid receptor type 1; CB2, cannabinoid receptor type 2; NAPE, N-acylphosphatidylethanolamine; NAPE-PLD, N-acylphosphatidylethanolamine-specific phospholipase D; DAG, diacylglycerol; DAGL, diacylglycerol lipase; FAAH, fatty acid amide hydrolase; MAGL, monoacylglycerol lipase.

CB1 was identified pharmacologically in 1988 and cloned in 1990, followed by the cloning of CB2 in 1993 (Devane et al., 1988; Matsuda et al., 1990; Munro et al., 1993). The subsequent identification of anandamide and 2-arachidonoylglycerol as endogenous cannabinoid ligands established the ECS as an endogenous signaling network involved in the regulation of pain, appetite, mood, memory, immune function, metabolism, inflammation, and cellular homeostasis (Devane et al., 1992; Di M et al., 2015; Zou and Kumar, 2018; Cristino et al., 2020).

CB1 and CB2 are class A G protein-coupled receptors that signal predominantly through Gi/o proteins and regulate intracellular pathways including MAPK/ERK and PI3K/Akt (Howl et al., 2002; Mackie, 2008). The two receptors exhibit distinct but overlapping tissue distributions (Figure 3) (Di M et al., 2015; Zou and Kumar, 2018; Cristino et al., 2020; Howl et al., 2002; Mackie, 2008; Ibeas Bih et al., 2015). CB1 is most abundant in the central nervous system but is also expressed in peripheral nerves, adipose tissue, and multiple peripheral organs (Di M et al., 2015; Zou and Kumar, 2018; Cristino et al., 2020; Howl et al., 2002; Mackie, 2008). In contrast, CB2 is predominantly associated with immune and hematopoietic tissues, including the spleen and bone marrow, with expression also reported in the lung, skin, gastrointestinal tract, and adipose tissue (Di M et al., 2015; Zou and Kumar, 2018; Cristino et al., 2020; Howl et al., 2002; Mackie, 2008; Ibeas Bih et al., 2015; Pertwee et al., 2010). Within the central nervous system, CB2 expression is relatively limited under basal conditions but may be enriched or upregulated in activated microglia and other glial cells during inflammatory states (Howl et al., 2002; Mackie, 2008; Ibeas Bih et al., 2015; Pertwee et al., 2010). Expression of both receptors varies according to cell type, physiologic state, and disease context (Howl et al., 2002; Mackie, 2008; Ibeas Bih et al., 2015; Pertwee et al., 2010).

**FIGURE 3:**
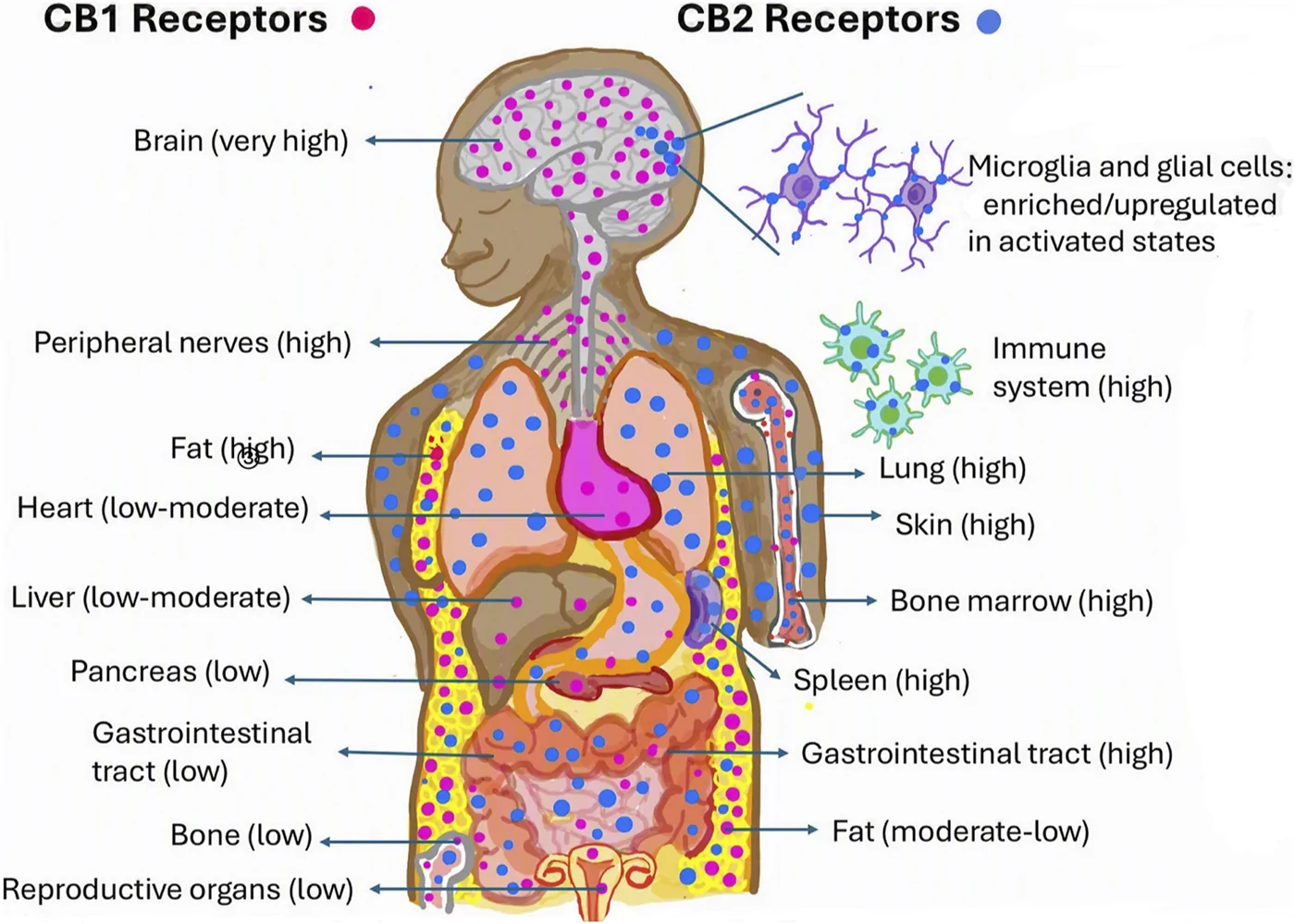
Tissue Distribution of CB1 and CB2 Receptors. CB1 receptors are widely distributed but are predominantly expressed in the central and peripheral nervous systems, whereas CB2 receptors are more prominently expressed in immune and hematopoietic tissues. The density of colored dots and the terms very high, high, moderate, and low provide a qualitative representation of relative receptor expression across tissues based on published tissue-distribution data and do not represent a quantitative expression scale. Basal CB2 expression in the central nervous system is relatively limited but may increase in activated microglia and other glial cells under inflammatory or pathological conditions (Zou and Kumar, 2018; Mackie, 2008; Maccarrone et al., 2015).

Beyond CB1 and CB2, cannabinoids interact with numerous noncanonical molecular targets, including transient receptor potential (TRP) channels, peroxisome proliferator-activated receptor-γ (PPARγ), G protein-coupled receptor 55 (GPR55), serotonin 5-HT1A receptors, adenosine signaling pathways, and voltage-gated ion channels (Ibeas Bih et al., 2015; Morales et al., 2017; Velasco et al., 2016; Ladin et al., 2016; Pertwee et al., 2010; Pertwee, 2008).

Unlike conventional neurotransmitters, endocannabinoids are synthesized on demand from membrane phospholipid precursors rather than stored in synaptic vesicles. Anandamide functions primarily as a partial agonist at CB1, whereas 2-arachidonoylglycerol is a full agonist at both CB1 and CB2 receptors. Endocannabinoid signaling is terminated principally through enzymatic degradation, with fatty acid amide hydrolase (FAAH) primarily metabolizing anandamide and monoacylglycerol lipase (MAGL) primarily metabolizing 2-arachidonoylglycerol (Di Marzo and Fontana, 1995; Piomelli, 2003; Lu and Mackie, 2016; Long and Cravatt, 2011).

Altered expression of CB1, CB2, GPR55, FAAH, MAGL, and other components of the ECS has been reported in glioblastoma and numerous solid and hematologic malignancies (Guzmán, 2003; Velasco et al., 2012). Experimental studies indicate that cannabinoid signaling can influence proliferation, apoptosis, autophagy, angiogenesis, epithelial–mesenchymal transition, invasion, metastasis, immune regulation, and tumor–microenvironment interactions through pathways including PI3K/Akt/mTOR, MAPK/ERK, EGFR, VEGF, NF-κB, and transforming growth factor-β signaling (Guzmán, 2003; Velasco et al., 2012; Velasco et al., 2016; Ladin et al., 2016; Dariš et al., 2019; Tomko et al., 2020). However, these findings derive predominantly from *in vitro* and animal models and have not translated into convincing clinical evidence of antitumor efficacy. Receptor expression alone does not reliably predict response to cannabinoid therapy, and both tumor-suppressive and tumor-promoting effects have been reported depending on tumor type, molecular subtype, receptor expression, cannabinoid exposure, and the tumor microenvironment (Guzmán, 2003; Velasco et al., 2012; Velasco et al., 2016; Ladin et al., 2016; Dariš et al., 2019; Tomko et al., 2020). The ECS should therefore be regarded as a context-dependent regulatory network rather than a uniformly tumor-suppressive pathway. Rigorous translational and clinical investigation is required before cannabinoid-based therapies can be incorporated into routine oncology practice. The available preclinical and clinical evidence across major malignancies is summarized in Table 3.

**TABLE 3:** Preclinical and clinical evidence for cannabinoids across major malignancies.

Tumor type	Main target/mechanism	Preclinical evidence represented in this review	Clinical evidence	References
Glioblastoma	CB1/CB2, TRPV2, TMZ synergy	*In vitro* and *in vivo* studies	Pilot/phase Ib; ongoing phase II investigation	(Guzmán et al., 2006; Twelves et al., 2021; López-Valero et al., 2018; Rocha et al., 2014; Bhaskaran et al., 2024; Nabissi et al., 2013)
Breast cancer	CBD, Id-1, Akt/EGFR	*In vitro* and *in vivo* studies	None established	(Caffarel et al., 2010; McAllister et al., 2007; McAllister et al., 2011; Elbaz et al., 2015)
Prostate cancer	CB1/TRPV1	*In vitro* studies; translational studies	Phase I CBD study	(Ramos and Bianco, 2012; Czifra et al., 2009; Myint et al., 2023)
Pancreatic cancer	GPR55, ER stress	*In vitro* and *in vivo* studies	None established	(Piñeiro et al., 2011; Sharafi et al., 2019; Ferro et al., 2018; Mangal et al., 2024)
NSCLC	CB1/CB2, COX-2/PPARγ	*In vitro* and *in vivo* studies	Case reports/limited observations	(Guggisberg et al., 2022; Preet et al., 2011; Ramer et al., 2013; Ramer et al., 2012)
Colorectal cancer	CB1, GPR55, CBG/CBC	*In vitro* and *in vivo* studies	None established	(Borrelli et al., 2014; Romano et al., 2013; Wang et al., 2008; Hasenoehrl et al., 2018)
Hepatocellular carcinoma	PPARγ	*In vitro* and *in vivo* studies	None established	(Vara et al., 2013; Afrin et al., 2020)
Head and neck SCC	Mixed/context-dependent signaling	*In vitro* studies	None established	(Nallathambi et al., 2023; Liu et al., 2020)
Gynecologic cancers	CBD/TRPV2, apoptosis	Predominantly *in vitro* studies	Observational data only; no established clinical efficacy	(Cao et al., 2024; Koltai and Shalev, 2022; Peleg et al., 2024; Marinelli et al., 2020; Lukhele and Motadi, 2016)
Hematologic malignancies	TRPV2, drug synergy	*In vitro* studies	None established	(Irrera et al., 2021; Morelli et al., 2014; Maggi et al., 2022)

The preclinical evidence column indicates the experimental model types represented in the studies cited in this review and is not intended as a quantitative or formal grading of evidence strength. Because this is a narrative review, the cited studies should not be interpreted as a comprehensive count of the available literature. No cannabinoid-based anticancer therapy has established clinical efficacy; all remain investigational.

Abbreviations: CBC, cannabichromene; CBG, cannabigerol; CBD, cannabidiol; CB1, cannabinoid receptor 1; CB2, cannabinoid receptor 2; COX-2, cyclooxygenase-2; EGFR, epidermal growth factor receptor; ER, endoplasmic reticulum; GPR55, G protein-coupled receptor 55; NSCLC, non-small cell lung cancer; PPARγ, peroxisome proliferator-activated receptor-γ; SCC, squamous cell carcinoma; TMZ, temozolomide; TRPV1/2, transient receptor potential vanilloid 1/2.

### 2.3 Clinical pharmacology

The clinical effects of cannabis are determined by cannabinoid composition, route of administration, dose, duration of exposure, prior cannabinoid use, concomitant medications, genetic variability in drug metabolism, and individual susceptibility (Grotenhermen, 2003). THC is the principal psychoactive constituent responsible for intoxication, whereas CBD lacks clinically meaningful intoxicating effects at therapeutic doses. Consequently, the efficacy, safety, and adverse-effect profile of cannabis products vary substantially according to their relative THC and CBD content (Pertwee et al., 2010; Pertwee, 2008; Grotenhermen, 2003).

Route of administration markedly influences cannabinoid pharmacokinetics. Inhaled cannabis is rapidly absorbed through the pulmonary circulation, producing clinical effects within minutes, with peak effects occurring after approximately 15–30 min and a typical duration of 2–4 h (Huestis, 2007; Grotenhermen, 2003; Lucas et al., 2018). This rapid onset facilitates symptom-guided dose titration but also increases the risk of acute intoxication, particularly with high-potency THC products. In contrast, orally administered cannabinoids undergo extensive first-pass hepatic metabolism, resulting in a delayed onset of approximately 30–120 min and clinical effects that may persist for 6–8 h or longer (Grotenhermen, 2003). Oral bioavailability is highly variable because of differences in gastrointestinal absorption, food intake, formulation, and hepatic metabolism. Consequently, cannabis-naïve individuals may administer additional doses prematurely, increasing the risk of excessive or prolonged intoxication (Huestis, 2007; Grotenhermen, 2003; Lucas et al., 2018).

THC is highly lipophilic, distributes rapidly into the brain, adipose tissue, and other highly perfused organs, and is metabolized primarily by CYP2C9 and CYP3A4 to the active metabolite 11-hydroxy-THC, which contributes substantially to the prolonged psychoactive effects observed after oral administration (Grotenhermen, 2003). CBD is metabolized predominantly through CYP2C19 and CYP3A4 and exhibits considerable pharmacokinetic variability related to formulation, dose, and route of administration (Zendulka et al., 2016; Grotenhermen, 2003; Lucas et al., 2018). These characteristics, together with differences in cannabinoid composition, contribute to substantial interindividual variability in therapeutic response and adverse effects.

Pharmaceutical cannabinoid formulations provide standardized composition, dosing, and quality control, whereas nonprescription cannabis products frequently demonstrate considerable variability in cannabinoid concentration, contaminants, and labeling accuracy, contributing to inconsistent therapeutic responses and potential adverse effects (Skórzewska and Gęca, 2024; Whiting et al., 2015; Braun et al., 2024; Rock and Parker, 2021).

The strongest clinical evidence supporting cannabinoid therapy relates to supportive care rather than direct anticancer treatment. Dronabinol and nabilone may benefit selected patients with chemotherapy-induced nausea and vomiting that persists despite guideline-directed antiemetic therapy (Braun et al., 2024; Herrstedt et al., 2024; National Comprehensive Cancer Network, 2026; U.S. Food and Drug Administration, 2017; U.S. Food and Drug Administration, 2024a; U.S. Food and Drug Administration, 2006). Evidence supporting cannabinoids for cancer-related pain, appetite loss, sleep disturbance, anxiety, and overall symptom burden remains modest and heterogeneous because of substantial differences in study design, cannabinoid formulations, dosing strategies, routes of administration, and patient populations (Whiting et al., 2015; Braun et al., 2024; Häuser et al., 2023).

## 3 Adverse effects

Although cannabinoids are often perceived as relatively safe, their adverse-effect profiles vary according to THC concentration, CBD content, dose, route of administration, duration of exposure, concomitant medications, and individual patient characteristics (Whiting et al., 2015; Braun et al., 2024; Huestis, 2007; Zendulka et al., 2016; U.S. Food and Drug Administration, 2024b; Cheah et al., 2024; Lucas et al., 2018; Kansagara et al., 2026). Most acute neuropsychiatric toxicity is attributable to THC, whereas CBD generally has a more favorable safety profile but may still cause somnolence, fatigue, gastrointestinal symptoms, decreased appetite, hepatic enzyme elevations, and clinically important pharmacokinetic interactions (Zendulka et al., 2016; U.S. Food and Drug Administration, 2024b; Millar et al., 2019; Cheah et al., 2024; Lucas et al., 2018). These considerations are particularly relevant in oncology because many patients are older, medically frail, have multiple comorbidities, or receive treatments with overlapping toxicities (Braun et al., 2024; Arkell et al., 2020; Kansagara et al., 2026).

### 3.1 Neuropsychiatric effects

Acute THC exposure may cause dizziness, somnolence, impaired attention, short-term memory dysfunction, altered sensory perception, slowed reaction time, impaired psychomotor performance, and reduced driving ability (Whiting et al., 2015; Huestis, 2007; Grotenhermen, 2003; Arkell et al., 2020; Lucas et al., 2018; Kansagara et al., 2026). THC exhibits a dose-dependent biphasic effect on anxiety, with lower doses potentially producing anxiolytic effects and higher doses more likely to precipitate anxiety, panic, paranoia, hallucinations, or transient psychotic symptoms, particularly in cannabis-naïve individuals and those with underlying psychiatric vulnerability (Whiting et al., 2015; Braun et al., 2024; Kansagara et al., 2026).

Frequent use of high-potency THC products has been associated with cognitive impairment, cannabis use disorder, and an increased risk of psychosis, particularly in adolescents, young adults, and other susceptible individuals (Whiting et al., 2015; Braun et al., 2024; Kansagara et al., 2026). Regular use of high-THC products in adolescents and young adults has been associated with an approximately 2- to 11-fold increased risk of psychosis (Kansagara et al., 2026). Cannabis may also worsen psychotic symptoms in individuals with schizophrenia or other psychotic-spectrum disorders and may exacerbate mania and impair functioning in those with bipolar disorder, whereas evidence regarding the long-term effects of THC-predominant cannabis on depression and anxiety remains less definitive (Braun et al., 2024; Kansagara et al., 2026). Although CBD may attenuate selected THC-related neuropsychiatric effects, it does not reliably prevent THC-induced cognitive impairment or psychomotor dysfunction (Ibeas Bih et al., 2015; Pertwee et al., 2010; Pertwee, 2008; Murray et al., 2024; Arkell et al., 2020).

In older adults with cancer, sedation, impaired balance, orthostatic hypotension, and cognitive dysfunction may increase the risk of falls, fractures, delirium, and functional decline, particularly when cannabinoids are combined with opioids, benzodiazepines, sedative-hypnotics, antihistamines, or other centrally acting medications (Braun et al., 2024; Cheah et al., 2024; Kansagara et al., 2026).

### 3.2 Cardiovascular and pulmonary effects

Acute THC exposure commonly causes tachycardia, peripheral vasodilation, orthostatic hypotension, and transient blood-pressure fluctuations (Whiting et al., 2015; Braun et al., 2024; Kansagara et al., 2026). Observational studies have also reported associations with myocardial infarction, particularly within the first hour after use, arrhythmias, ischemic stroke, and heart-failure exacerbation, although causality remains uncertain because of confounding factors such as tobacco exposure, route of administration, and cannabis potency (Whiting et al., 2015; Braun et al., 2024; Kansagara et al., 2026). Caution is therefore warranted in patients with significant cardiovascular disease.

Regular cannabis smoking is associated with chronic cough, sputum production, bronchitic symptoms, and airway inflammation (National Academies of Sciences, 2017; Kansagara et al., 2026). Although an independent association with lung cancer has not been conclusively established, cannabis smoke contains many of the same combustion products and carcinogens as tobacco smoke (National Academies of Sciences, 2017; Whiting et al., 2015; Kansagara et al., 2026).

Smoked or unregulated cannabis may expose immunocompromised patients to fungal contamination, including *Aspergillus* species, and cases of aspergillosis associated with cannabis use have been reported in immunocompromised individuals (National Academies of Sciences, 2017). Consequently, patients receiving intensive chemotherapy, hematopoietic stem-cell transplantation, prolonged corticosteroids, or other immunosuppressive therapies should avoid smoked or unregulated inhaled cannabis products. When cannabinoid therapy is appropriate, regulated oral or pharmaceutical formulations are generally preferred (National Academies of Sciences, 2017; Braun et al., 2024).

### 3.3 Gastrointestinal and hepatic effects

Cannabinoid hyperemesis syndrome (CHS) is an increasingly recognized complication of chronic, frequent cannabis use and is characterized by recurrent nausea, vomiting, abdominal pain, and compulsive hot bathing or showering that temporarily relieves symptoms (Braun et al., 2024; Alderman et al., 2023; Kansagara et al., 2026). Because affected patients often continue cannabis use in an attempt to control nausea, the syndrome may be mistaken for chemotherapy-induced nausea and vomiting, gastrointestinal obstruction, medication toxicity, or progressive malignancy. Sustained cessation of cannabis remains the most effective treatment (Braun et al., 2024; Alderman et al., 2023).

CBD may also cause diarrhea, nausea, decreased appetite, weight loss, and hepatic enzyme elevations, particularly at higher pharmaceutical doses (Zendulka et al., 2016; U.S. Food and Drug Administration, 2024b; Millar et al., 2019). These adverse effects may be clinically important in patients with pre-existing anorexia, cachexia, treatment-related diarrhea, poor nutritional status, or underlying liver disease.

### 3.4 Reproductive and developmental effects

THC crosses the placenta and is excreted into breast milk. Cannabis use during pregnancy and lactation is therefore discouraged because of concerns regarding impaired fetal growth, altered neurodevelopment, maternal cognitive impairment, and prolonged infant exposure (National Academies of Sciences, 2017; Braun et al., 2024; Arkell et al., 2020; Kansagara et al., 2026). Chronic cannabis use has also been associated with adverse effects on male and female reproductive function, including impaired spermatogenesis and altered hypothalamic-pituitary-gonadal signaling, potentially disrupting ovulation and reducing fertility, although the magnitude and reversibility of these effects remain uncertain (National Academies of Sciences, 2017; Arkell et al., 2020; Kansagara et al., 2026).

### 3.5 Dependence, cannabis use disorder, and withdrawal

Repeated exposure to high-potency THC products may result in tolerance, physical dependence, and cannabis use disorder, particularly with frequent, prolonged use and early initiation (National Academies of Sciences, 2017; Whiting et al., 2015; Kansagara et al., 2026). Abrupt discontinuation may produce a withdrawal syndrome characterized by irritability, anxiety, insomnia, disturbed dreaming, depressed mood, restlessness, decreased appetite, and abdominal discomfort. Symptoms generally begin within 24–72 h, peak during the first week, and resolve over 2–4 weeks, although sleep disturbance and craving may persist longer in heavy users (National Academies of Sciences, 2017; Kansagara et al., 2026).

Although cannabis dependence is generally less medically hazardous than dependence on alcohol, benzodiazepines, or opioids, it may nevertheless cause clinically significant psychological, occupational, and social impairment. Long-term cannabinoid therapy should therefore include periodic assessment for cannabis use disorder, particularly in patients requiring escalating doses or reporting impaired control or withdrawal symptoms (Whiting et al., 2015; Braun et al., 2024; Kansagara et al., 2026).

### 3.6 Considerations in patients with cancer

The clinical significance of cannabinoid toxicity extends beyond direct adverse effects. Patients with cancer frequently receive therapies associated with neurological, cardiovascular, hepatic, gastrointestinal, or hematologic toxicities, and cannabinoids may further increase these risks through additive toxicity or pharmacokinetic interactions. Careful patient selection, medication reconciliation, assessment of frailty and fall risk, and ongoing clinical monitoring are therefore essential when cannabinoids are incorporated into supportive cancer care (Braun et al., 2024; Zendulka et al., 2016; Cheah et al., 2024; Arkell et al., 2020; Lucas et al., 2018; Kansagara et al., 2026).

## 4 Drug interactions

Cannabinoids have substantial potential for pharmacokinetic and pharmacodynamic drug interactions because THC and CBD undergo extensive hepatic metabolism and may modulate several cytochrome P450 (CYP) enzymes, uridine 5′-diphospho-glucuronosyltransferases (UGTs), and, based largely on *in vitro* evidence, selected drug transporters (Huestis, 2007; Zendulka et al., 2016; U.S. Food and Drug Administration, 2024b; Grotenhermen, 2003; Lucas et al., 2018; Qian et al., 2019; Buchtova et al., 2023; Leino et al., 2019). These interactions are particularly relevant in oncology, where patients frequently receive multiple concomitant medications, including oral targeted therapies, anticoagulants, antiepileptic drugs, immunosuppressants, opioids, and supportive-care agents with narrow therapeutic indices or concentration-dependent toxicities (Braun et al., 2024; Zendulka et al., 2016; Qian et al., 2019; Buchtova et al., 2023).

### 4.1 Pharmacokinetic mechanisms

THC is metabolized predominantly by CYP2C9 and CYP3A4, whereas CBD is metabolized primarily by CYP2C19 and CYP3A4 (Huestis, 2007; Zendulka et al., 2016; Grotenhermen, 2003; Lucas et al., 2018; Qian et al., 2019). Cannabinoids may inhibit CYP2C9 and CYP2C19 at clinically relevant exposures, whereas inhibition of CYP3A4, CYP2D6, CYP2B6, UGT enzymes, and drug transporters is supported mainly by *in vitro* or limited clinical evidence (Zendulka et al., 2016; Qian et al., 2019; Buchtova et al., 2023). The magnitude of interaction depends on cannabinoid dose, formulation, route of administration, duration of exposure, and the therapeutic index of the concomitant medication. Pharmaceutical doses of CBD are more likely to produce clinically significant interactions than the lower or inconsistently absorbed doses found in many nonprescription products (Zendulka et al., 2016; U.S. Food and Drug Administration, 2024b; Millar et al., 2019; Lucas et al., 2018; Qian et al., 2019).

Conversely, medications that alter CYP activity may influence cannabinoid exposure. Strong CYP3A4 or CYP2C19 inhibitors may increase CBD concentrations, whereas potent enzyme inducers—including rifampin, carbamazepine, phenytoin, phenobarbital, and *Hypericum perforatum* (St. John’s wort)—may reduce cannabinoid exposure and diminish clinical effects (Zendulka et al., 2016; U.S. Food and Drug Administration, 2024b; Lucas et al., 2018; Qian et al., 2019). Strong CYP2C9 inhibitors may similarly increase THC concentrations. Clinicians should therefore consider these interactions bidirectional.

### 4.2 Clinically important drug interactions

One of the best-characterized interactions involves warfarin. CBD-mediated inhibition of CYP2C9 may reduce warfarin clearance, increase the international normalized ratio (INR), and elevate bleeding risk (Zendulka et al., 2016; Qian et al., 2019). Although evidence is derived primarily from case reports and small observational studies, the narrow therapeutic index of warfarin warrants closer INR monitoring whenever cannabinoid therapy is initiated, discontinued, or substantially modified.

Clinically important interactions have also been demonstrated with calcineurin inhibitors. Pharmaceutical-dose CBD has produced approximately threefold increases in dose-normalized tacrolimus concentrations, and a subsequent controlled pharmacokinetic study confirmed increased tacrolimus exposure during CBD administration (Leino et al., 2019; So et al., 2025). Concomitant use may increase the risk of nephrotoxicity, neurotoxicity, hypertension, electrolyte abnormalities, and other concentration-dependent adverse effects. Tacrolimus trough concentrations, renal function, and clinical toxicity should therefore be monitored closely when CBD is initiated, titrated, or discontinued. Similar caution is appropriate for cyclosporine and other CYP3A substrates with narrow therapeutic indices, although direct clinical evidence remains limited.

CBD may also alter the concentrations of several antiseizure medications. The best-established interaction involves clobazam, in which CBD inhibits CYP2C19 and reduces the metabolism of its active metabolite, N-desmethylclobazam. This increases systemic exposure and the risk of somnolence and sedation (U.S. Food and Drug Administration, 2024b; Qian et al., 2019).

Concomitant use with valproate has been associated primarily with hepatic aminotransferase elevations rather than a clearly defined pharmacokinetic interaction. These considerations are particularly relevant in patients with primary or metastatic brain tumors receiving antiseizure therapy.

Cannabis smoking introduces an additional interaction because polycyclic aromatic hydrocarbons generated during combustion induce CYP1A2. Chronic smoking may reduce exposure to CYP1A2 substrates such as clozapine, olanzapine, theophylline, and tizanidine, whereas abrupt smoking cessation may reverse enzyme induction and increase drug concentrations (Qian et al., 2019). This interaction results from combustion products rather than THC itself and is not expected with oral cannabinoid formulations.

The major established and potential interactions between cannabis or cannabinoids and commonly used medications are summarized in Table 4.

**TABLE 4:** Clinically important drug interactions with cannabis and cannabinoids.

Drug/class	Primary mechanism	Potential clinical consequences	Clinical recommendation
Warfarin	CBD inhibits CYP2C9	Increased INR and bleeding risk^a^	Monitor INR closely after cannabinoid initiation, discontinuation, or substantial dose changes
Direct oral anticoagulants (DOACs)	Potential CYP3A4 and/or P-gp inhibition by CBD	Potential increased anticoagulant exposure and bleeding risk; clinical evidence limited^a^	Use cautiously; monitor for bleeding, particularly with high-dose CBD.
Tacrolimus	CBD inhibits CYP3A4 and may modulate P-gp–mediated transport	Increased tacrolimus exposure; nephrotoxicity, neurotoxicity, and other concentration-dependent adverse effects^b^	Monitor tacrolimus trough concentrations and renal function closely; adjust tacrolimus dose as needed
Cyclosporine	Potential CYP3A4 and P-gp inhibition by CBD	Potential increased cyclosporine exposure and toxicity; direct clinical evidence limited^a^	Monitor trough concentrations, renal function, and clinical toxicity
Opioids	Additive CNS-depressant effects	Increased sedation, cognitive impairment, and falls; potential respiratory depression with other CNS depressants^c^	Use cautiously; monitor sedation and respiratory status in high-risk patients
Benzodiazepines and sedative-hypnotics	Additive CNS-depressant effects	Excessive sedation, impaired cognition and coordination, and falls^c^	Use cautiously; minimize concurrent sedative burden and monitor clinically
Alcohol	Additive CNS and psychomotor impairment	Increased intoxication, impaired coordination and judgment, and accident risk^d^	Avoid or minimize concurrent use
Antidepressants (SSRIs, TCAs)	CBD may inhibit CYP2C19, CYP2D6, and CYP3A4	Potential increased drug exposure and adverse effects; magnitude varies by agent^a^	Monitor for adverse effects, particularly with higher CBD doses and susceptible CYP substrates
Antipsychotics (e.g., clozapine, olanzapine)	Cannabis smoke induces CYP1A2; CBD may inhibit selected CYP enzymes	Smoking may reduce concentrations of CYP1A2 substrates; smoking cessation may increase concentrations and toxicity^a^	Monitor clinical response and drug concentrations when feasible after changes in cannabis-smoking habits
Antiseizure medications (especially clobazam)	CBD inhibits CYP2C19, increasing N-desmethylclobazam exposure	Increased sedation and other concentration-dependent adverse effects^e^	Monitor for sedation; consider dose adjustment and therapeutic drug monitoring when appropriate
Strong CYP inhibitors (e.g., azole antifungals, clarithromycin, ritonavir)	Reduced CYP-mediated cannabinoid metabolism	Increased THC and/or CBD exposure and cannabinoid-related toxicity^f^	Monitor for cannabinoid adverse effects; consider dose reduction when clinically indicated
Strong enzyme inducers (e.g., rifampin, carbamazepine, phenytoin, phenobarbital, St. John’s wort)	Increased cannabinoid metabolism	Reduced cannabinoid exposure and diminished clinical effect^f^	Monitor clinical response; avoid empiric cannabinoid dose escalation without reassessment
Immune checkpoint inhibitors	Possible cannabinoid-mediated immunomodulation	Potential alteration of ICI efficacy; available clinical evidence is conflicting and causality is unproven^g^	Document cannabinoid exposure and discuss uncertainty; routine avoidance is not supported by current evidence
Tamoxifen	CBD may inhibit CYP2D6 and CYP3A4	Potential reduction in formation/exposure of active metabolite endoxifen^h^	Use pharmacological-dose CBD cautiously; consider potential interaction, particularly in patients with reduced CYP2D6 activity
Oral targeted therapies (e.g., selected EGFR, ALK, BCR-ABL, BTK, CDK4/6, BRAF, MEK, VEGFR, and PARP inhibitors)	Potential inhibition of CYP3A4 and other metabolic/transport pathways by CBD	Potential increased drug exposure and concentration-dependent toxicity; clinical evidence remains limited^i^	Review the metabolic pathway of the specific agent; monitor for toxicity and laboratory abnormalities; avoid unsupported empiric dose adjustment

References.

^a^ Refs. (Zendulka et al., 2016; Qian et al., 2019).

^b^ Refs. (Leino et al., 2019; So et al., 2025).

^c^ Refs. (Braun et al., 2024; Huestis, 2007).

^d^ Refs. (Huestis, 2007; Huestis, 2002).

^e^ Refs. (U.S., food and drug administration, 2024b; Qian et al., 2019).

^f^ Refs. (Huestis, 2007; Zendulka et al., 2016; Qian et al., 2019).

^g^ Refs. (Taha et al., 2019; Bar-Sela et al., 2020; Waissengrin et al., 2023; Behling-Hess et al., 2025).

^h^ Ref. (Parihar et al., 2022).

^i^ Refs. (Zendulka et al., 2016; Qian et al., 2019; Buchtova et al., 2023).

Abbreviations: ALK, anaplastic lymphoma kinase; BCR-ABL, breakpoint cluster region–Abelson; BRAF, B-Raf proto-oncogene; BTK, bruton tyrosine kinase; CBD, cannabidiol; CDK4/6, cyclin-dependent kinase 4/6; CNS, central nervous system; CYP, cytochrome P450; DOAC, direct oral anticoagulant; EGFR, epidermal growth factor receptor; ICI, immune checkpoint inhibitor; INR, international normalized ratio; MEK, mitogen-activated protein kinase kinase; PARP, poly(ADP-ribose) polymerase; P-gp, P-glycoprotein; SSRI, selective serotonin reuptake inhibitor; TCA, tricyclic antidepressant; THC, Δ^9^-tetrahydrocannabinol; VEGFR, vascular endothelial growth factor receptor.

### 4.3 Pharmacodynamic interactions

Pharmacodynamic interactions are equally important. Concomitant use of THC-containing products with opioids, benzodiazepines, sedative-hypnotics, antihistamines, gabapentinoids, alcohol, or other central nervous system depressants may increase somnolence, confusion, impaired coordination, falls, and psychomotor dysfunction (Whiting et al., 2015; Braun et al., 2024; Murray et al., 2024; Häuser et al., 2018; Kansagara et al., 2026). These effects are particularly consequential in older or frail patients with cancer and in those with brain metastases, baseline cognitive impairment, autonomic dysfunction, or increased fall risk.

### 4.4 Interactions with anticancer therapy

Potential interactions between cannabinoids and anticancer therapies remain clinically important but incompletely characterized. Numerous oral targeted therapies—including selected EGFR, ALK, BCR-ABL, BTK, CDK4/6, BRAF, MEK, VEGFR, and PARP inhibitors—are metabolized through CYP3A4, CYP2C8, CYP2C9, or drug transporters. Pharmacological-dose CBD could theoretically increase exposure to susceptible agents and exacerbate concentration-dependent toxicities, including hepatotoxicity, myelosuppression, diarrhea, mucositis, QT-interval prolongation, hypertension, bleeding, and dermatologic toxicity (Zendulka et al., 2016; Qian et al., 2019; Buchtova et al., 2023). However, most proposed interactions are based on metabolic pathways, *in vitro* studies, or extrapolation from nononcologic drugs rather than prospective clinical pharmacokinetic investigations.

Routine empiric dose adjustment of targeted therapies solely because of cannabinoid use cannot currently be recommended because clinically relevant interactions have not been established in prospective pharmacokinetic or clinical studies (Qian et al., 2019; Buchtova et al., 2023). Nevertheless, clinicians should consider a cannabinoid interaction when otherwise unexplained toxicity develops after cannabinoid initiation or dose escalation, or when treatment efficacy or toxicity changes following cannabinoid discontinuation. Temporary interruption of cannabinoid therapy, therapeutic drug monitoring when available, and consultation with an oncology pharmacist may help clarify the relationship.

Potential interactions with endocrine therapy also warrant consideration. Tamoxifen is converted to its active metabolite, endoxifen, predominantly through CYP2D6, with additional contributions from CYP3A4. CBD inhibits these enzymes *in vitro*, and a case report described reduced endoxifen and N-desmethyltamoxifen concentrations during chronic low-dose CBD administration, with recovery following CBD discontinuation (Parihar et al., 2022). Although the clinical significance remains uncertain, pharmacological-dose CBD should be used cautiously in patients receiving tamoxifen, particularly those with reduced CYP2D6 activity.

### 4.5 Immune checkpoint inhibitors

The relationship between cannabinoids and immune checkpoint inhibitor (ICI) efficacy has attracted considerable attention because cannabinoids possess anti-inflammatory and immunomodulatory properties that could theoretically attenuate antitumor immunity (Guzmán, 2003; Velasco et al., 2012; Velasco et al., 2016; Ladin et al., 2016). Early retrospective studies reported lower response rates, shorter progression-free survival, or shorter overall survival among cannabis users receiving PD-1 or PD-L1 inhibitors (Taha et al., 2019; Bar-Sela et al., 2020). However, these studies were limited by small sample size, heterogeneous cancer types and cannabis products, incomplete characterization of cannabinoid exposure, and potential confounding by symptom burden and disease severity.

Subsequent investigations have not consistently reproduced these findings. A prospective observational study of patients with non-small cell lung cancer receiving pembrolizumab found no significant adverse effect of medical cannabis on treatment efficacy or survival (Waissengrin et al., 2023). Likewise, recent systematic reviews concluded that current evidence remains heterogeneous and insufficient to determine whether cannabis either compromises or preserves ICI efficacy (Behling-Hess et al., 2025).

Current evidence does not support routine avoidance of cannabinoids during ICI therapy. Patients should nevertheless be informed that clinically meaningful interactions remain uncertain, and cannabinoid exposure should be documented carefully, including product type, THC-to-CBD ratio, dose, route, frequency, timing relative to immunotherapy, and indication for use. Cannabis should not be initiated with the expectation of improving immunotherapy efficacy.

### 4.6 Clinical considerations

Comprehensive medication reconciliation should include prescription cannabinoids, botanical cannabis, CBD oils, edibles, inhaled products, and other hemp-derived preparations. Particular vigilance is warranted in patients receiving medications with narrow therapeutic indices, therapeutic drug monitoring requirements, clinically important CYP2C9-, CYP2C19-, CYP3A4-, or CYP2D6-mediated metabolism, or overlapping neurological, hepatic, cardiovascular, hematologic, or sedative toxicities (Braun et al., 2024; Zendulka et al., 2016; Lucas et al., 2018; Qian et al., 2019; Buchtova et al., 2023; Leino et al., 2019; So et al., 2025). Because available evidence remains incomplete, management should emphasize individualized risk assessment, close clinical and laboratory monitoring, and avoidance of unsupported empiric dose adjustments.

## 5 Cannabinoids in supportive oncology

Although cannabinoids remain investigational as anticancer therapies, their role in supportive oncology has expanded substantially. Patients with cancer commonly use cannabis or cannabinoid products to alleviate chemotherapy-induced nausea and vomiting (CINV), pain, appetite loss, insomnia, anxiety, and overall symptom burden (Pergam et al., 2017; Braun et al., 2024; Fallon et al., 2017; Johnson et al., 2010; Jatoi et al., 2002; Strasser et al., 2006; Duran et al., 2010; Grimison et al., 2020; Grimison et al., 2024; National Cancer Institute, 2026). Surveys indicate that many patients use cannabis during active cancer treatment without informing their oncology team, increasing the risk of unrecognized adverse effects and drug interactions (Pergam et al., 2017; Braun et al., 2018; Braun et al., 2024). Consequently, oncologists should be familiar with the current evidence, limitations, and professional society recommendations regarding cannabinoid use in supportive care.

### 5.1 Chemotherapy-induced nausea and vomiting

The strongest evidence supporting cannabinoid therapy in oncology relates to CINV. Randomized clinical trials conducted before the introduction of modern antiemetic regimens demonstrated that dronabinol and nabilone were superior to placebo and comparable to older dopamine-receptor antagonists for refractory CINV (Whiting et al., 2015; Smith et al., 2015). More recent randomized studies have also evaluated standardized THC:CBD preparations in patients with refractory CINV (Duran et al., 2010; Grimison et al., 2020). Both dronabinol and nabilone subsequently received regulatory approval for CINV inadequately controlled by conventional antiemetic therapy (U.S. Fo od and Drug Administration, 2017; U.S. Food and Drug Administration, 2024a; U.S. Food and Drug Administration, 2006).

Modern antiemetic regimens incorporating serotonin (5-HT3) receptor antagonists, neurokinin-1 (NK1) receptor antagonists, dexamethasone, and olanzapine provide substantially better symptom control than historical comparators (Braun et al., 2024; Herrstedt et al., 2024; National Comprehensive Cancer Network, 2026). Current oncology guidelines therefore reserve cannabis and cannabinoid-based therapies for selected patients with persistent or refractory CINV despite optimized standard antiemetic treatment. ASCO recognizes that cannabis and/or cannabinoids may improve refractory CINV when added to guideline-directed antiemetic regimens, whereas dronabinol and nabilone remain the best-established pharmaceutical cannabinoid options because of their standardized formulations, regulatory approval, and historical clinical-trial evidence (Braun et al., 2024; Herrstedt et al., 2024; National Comprehensive Cancer Network, 2026; U.S. Food and Drug Administration, 2017; U.S. Food and Drug Administration, 2024a; U.S. Food and Drug Administration, 2006; Smith et al., 2015).

### 5.2 Cancer-related pain

Cannabinoids modulate nociceptive processing through central, peripheral, and inflammatory mechanisms involving CB1, CB2, and non-cannabinoid targets, providing a biological rationale for analgesic activity (Howl et al., 2002; Mackie, 2008; Ibeas Bih et al., 2015; Morales et al., 2017; Velasco et al., 2016; Ladin et al., 2016; Dariš et al., 2019; Tomko et al., 2020; Pertwee et al., 2010; Pertwee, 2008). Although some clinical studies have reported improvements in pain intensity, sleep quality, or overall symptom burden, randomized trials of THC:CBD preparations have yielded inconsistent results, with several failing to meet their primary efficacy endpoints (Whiting et al., 2015; Braun et al., 2024; Häuser et al., 2023; Fallon et al., 2017; Johnson et al., 2010). Contemporary systematic reviews and meta-analyses have not demonstrated a clinically meaningful analgesic benefit of cannabinoids for cancer-related pain (Table 5) (Braun et al., 2024; Häuser et al., 2023). Similarly, recent meta-analytic evidence has not demonstrated a consistent or clinically meaningful opioid-sparing effect in patients with cancer pain (Creangă-Murariu et al., 2026). Accordingly, current evidence does not support cannabinoids as standard therapy for cancer-related pain, although their use may be considered on an individualized basis for selected patients with refractory symptoms after evidence-based analgesic strategies have been optimized (Bhaskar et al., 2021; Braun et al., 2024).

**TABLE 5:** Selected clinical studies of cannabinoids in oncology.

Study	Setting/design	N	Cannabinoid, dose, and route	Comparator	Key outcome	Main limitation
(Grimison et al., 2024)	Refractory CINV; phase II/III RCT	147	THC 2.5 mg + CBD 2.5 mg, oral, 3×/day	Placebo	CR 24% vs. 8% (P = 0.01)	Limited use of olanzapine
(Häuser et al., 2023)	Cancer pain; Cochrane review	1,823	Mainly nabiximols/THC; predominantly oromucosal	Placebo/active control	No clinically meaningful analgesic benefit; pooled SMD −0.19 (95% CI −0.40 to 0.02)	Heterogeneous formulations and studies
(Guzmán et al., 2006)	Recurrent GBM; pilot study	9	THC, intratumoral	None	Feasible; biological activity observed; efficacy not established	Very small, uncontrolled
(Twelves et al., 2021)	Recurrent GBM; phase Ib randomized study	21*	Nabiximols (∼1:1 THC:CBD), oromucosal + TMZ	Placebo + TMZ	6-mo PFS 33% vs. 33%; 1-y OS 83% vs. 44%	Small; not powered for efficacy
(Taha et al., 2019)	Advanced cancers receiving nivolumab; retrospective	140	Medical cannabis; heterogeneous	Nivolumab alone	RR 15.9% vs. 37.5%; no significant PFS/OS difference	Observational; heterogeneous exposure
(Bar-Sela et al., 2020)	Metastatic cancers receiving ICIs; prospective observational	102	Medical cannabis; variable formulations/routes	ICI without cannabis	TTP 3.4 vs. 13.1 mo; OS 6.4 vs. 28.5 mo	Nonrandomized; confounding

* Twenty-one patients were included in the randomized phase (12 nabiximols; 9 placebo).

Abbreviations: CBD, cannabidiol; CINV, chemotherapy-induced nausea and vomiting; CR, complete response; GBM, glioblastoma; ICI, immune checkpoint inhibitor; OS, overall survival; PFS, progression-free survival; RCT, randomized controlled trial; RR, response rate; SMD, standardized mean difference; THC, Δ^9^-tetrahydrocannabinol; TMZ, temozolomide; TTP, time to progression.

### 5.3 Anorexia, cachexia, and appetite stimulation

THC stimulates appetite primarily through hypothalamic CB1 receptor activation, providing a biological rationale for its use in cancer-associated anorexia (Howl et al., 2002; Dariš et al., 2019; Pertwee et al., 2010; Pertwee, 2008). Clinical trials have demonstrated modest improvements in appetite and food enjoyment in some patients but have not consistently improved body weight, lean body mass, functional status, or survival (Whiting et al., 2015; Braun et al., 2024; Jatoi et al., 2002; Strasser et al., 2006).

Comparative trials have shown megestrol acetate to be superior to dronabinol for appetite stimulation and weight gain (Jatoi et al., 2002). Cannabinoids should therefore not replace nutritional assessment, dietary counseling, exercise interventions, or established pharmacologic therapies but may be considered for individualized symptomatic management in selected patients (Braun et al., 2024; Jatoi et al., 2002; Strasser et al., 2006).

### 5.4 Anxiety, sleep disturbance, and quality of life

CBD has demonstrated anxiolytic properties in experimental models and early clinical studies, whereas THC has dose-dependent, biphasic effects, potentially promoting relaxation and sedation at lower doses but anxiety, panic, paranoia, or dysphoria at higher doses (Ibeas Bih et al., 2015; Pertwee et al., 2010; Pertwee, 2008; Arkell et al., 2020; Kansagara et al., 2026). Observational studies frequently report improvements in anxiety, sleep quality, and overall quality of life; however, randomized controlled trials remain limited and have produced inconsistent results (Pergam et al., 2017; Braun et al., 2018; Braun et al., 2024; Kansagara et al., 2026).

Cannabinoids should therefore be regarded as adjunctive rather than primary therapy for anxiety, insomnia, or quality-of-life improvement. Careful patient selection should include assessment of psychiatric history, cognitive impairment, frailty, cardiovascular disease, fall risk, and concomitant sedating medications (Braun et al., 2024; Arkell et al., 2020; Kansagara et al., 2026).

### 5.5 Palliative and end-of-life care

Cannabinoids may be considered in selected patients receiving palliative or end-of-life care for symptom management, including pain, nausea, anorexia, anxiety, insomnia, and impaired quality of life, although evidence of benefit varies substantially across symptom domains (Braun et al., 2024; National Cancer Institute, 2026). Even modest symptomatic improvement may be meaningful when the primary therapeutic goal is comfort rather than disease modification. Treatment decisions should therefore be individualized after discussion of expected benefits, adverse effects, potential drug interactions, and the limitations of current evidence (Braun et al., 2024; National Cancer Institute, 2026).

### 5.6 Current guideline recommendations

Current oncology and supportive-care guidelines recognize a limited role for cannabis and cannabinoid-based therapies in supportive cancer care. ASCO indicates that cannabis and/or cannabinoids may improve refractory CINV when used as adjuncts to guideline-directed antiemetic therapy, while dronabinol and nabilone remain established pharmaceutical options for patients with CINV inadequately controlled by conventional treatment (Braun et al., 2024; Herrstedt et al., 2024; National Comprehensive Cancer Network, 2026). Evidence remains insufficient to recommend routine cannabinoid therapy for cancer-related pain, anorexia-cachexia syndrome, anxiety, insomnia, or quality-of-life improvement, although individualized use may be appropriate in selected patients after careful discussion of potential benefits, adverse effects, and uncertainty (Braun et al., 2024).

Importantly, no major oncology society recommends cannabis or cannabinoid products as cancer-directed therapy outside a well-designed clinical trial (Braun et al., 2024). Cannabinoids should therefore be regarded primarily as adjunctive supportive-care agents rather than substitutes for evidence-based symptom management or anticancer treatment. Their use should be individualized according to symptom burden, treatment goals, patient preferences, comorbidities, frailty, susceptibility to adverse effects, potential drug interactions, and product quality (Braun et al., 2024; Zendulka et al., 2016; Arkell et al., 2020; Lucas et al., 2018; Kansagara et al., 2026).

## 6 Potential anticancer activity of cannabinoids

### 6.1 Overview

The potential antitumor activity of cannabinoids has become an increasingly important area of cancer research. THC, CBD, endocannabinoids, synthetic cannabinoid receptor agonists, and several minor phytocannabinoids have demonstrated antiproliferative and cytotoxic activity in experimental models of glioma, breast, prostate, pancreatic, colorectal, lung, and other malignancies (Guzmán, 2003; Velasco et al., 2012; Velasco et al., 2016; Ladin et al., 2016; Dariš et al., 2019; Tomko et al., 2020; Guggisberg et al., 2022). However, clinical evidence remains limited to small early-phase trials, observational studies, and isolated case reports, and no cannabinoid formulation has demonstrated reproducible improvements in progression-free or overall survival in adequately powered randomized clinical trials (Braun et al., 2024; Guggisberg et al., 2022; Guzmán et al., 2006; Twelves et al., 2021).

Experimental studies indicate that cannabinoids influence multiple hallmarks of cancer, including cell proliferation, apoptosis, autophagy, angiogenesis, epithelial–mesenchymal transition, invasion, metastasis, oxidative stress, and tumor–microenvironment interactions through CB1-, CB2-, and noncanonical signaling pathways involving TRP channels, GPR55, PPARγ, PI3K/Akt/mTOR, MAPK/ERK, EGFR, VEGF, NF-κB, ceramide synthesis, mitochondrial dysfunction, reactive oxygen species generation, and endoplasmic-reticulum stress (Guzmán, 2003; Velasco et al., 2012; Ibeas Bih et al., 2015; Morales et al., 2017; Velasco et al., 2016; Ladin et al., 2016; Dariš et al., 2019; Tomko et al., 2020). The relative contribution of these mechanisms varies according to cannabinoid, tumor type, receptor expression, drug concentration, duration of exposure, and experimental model.

Among phytocannabinoids, CBD has attracted particular interest because of its broad receptor-dependent and receptor-independent biological activity and lack of classical intoxicating effects. Experimental studies have demonstrated modulation of oxidative stress, apoptosis, autophagy, migration, invasion, and multiple oncogenic signaling pathways, although many *in vitro* studies employed concentrations exceeding those achievable with clinically tolerated dosing (Ibeas Bih et al., 2015; Ladin et al., 2016; Dariš et al., 2019; Tomko et al., 2020; Caffarel et al., 2010; McAllister et al., 2007; McAllister et al., 2011; Elbaz et al., 2015).

Cannabinoids have also been investigated in combination with conventional anticancer therapies. Preclinical studies, particularly in glioblastoma, suggest that selected THC:CBD combinations may enhance the activity of temozolomide, whereas interactions with chemotherapy, radiotherapy, targeted therapy, and immunotherapy appear highly dependent on drug sequence, timing, cannabinoid formulation, ratio, and tumor type (Buchtova et al., 2023; López-Valero et al., 2018).

Overall, cannabinoid signaling is highly context dependent and may produce antitumor, neutral, or even tumor-promoting effects depending on cancer type, molecular subtype, receptor expression, cannabinoid composition, dose, route of administration, duration of exposure, immune status, and the tumor microenvironment (Guzmán, 2003; Velasco et al., 2012; Velasco et al., 2016; Ladin et al., 2016; Dariš et al., 2019; Tomko et al., 2020; Piñeiro et al., 2011). Accordingly, botanical cannabis, standardized extracts, purified phytocannabinoids, endocannabinoids, and synthetic cannabinoid receptor agonists should not be regarded as pharmacologically interchangeable. Current evidence supports cannabinoids as investigational anticancer agents rather than established cancer therapies, and they should not replace evidence-based oncologic treatment outside prospective clinical trials (Braun et al., 2024; Guggisberg et al., 2022).

The major proposed antitumor mechanisms of cannabinoids and their potential biological consequences are summarized in Table 6.

**TABLE 6:** Major proposed antitumor mechanisms of cannabinoids.

Biological process	Principal molecular targets/pathways	Major cellular Effects	Potential antitumor consequences	Representative references
Cell survival and proliferation	PI3K/Akt/mTOR, MAPK/ERK, EGFR, GPR55	↓ Proliferation and prosurvival signaling	Reduced tumor-cell growth	(Guzmán, 2003; Velasco et al., 2012; Velasco et al., 2016; Ladin et al., 2016; Dariš et al., 2019; Tomko et al., 2020; Caffarel et al., 2010; Elbaz et al., 2015; Piñeiro et al., 2011; Ferro et al., 2018; Preet et al., 2011)
Cell-cycle regulation	Cyclins/CDKs, p21, p27	Cell-cycle arrest	Reduced tumor-cell proliferation	(Guzmán, 2003; Velasco et al., 2012; Velasco et al., 2016; Ladin et al., 2016; Dariš et al., 2019; Tomko et al., 2020)
Apoptosis	Bax/Bcl-2 balance, caspases, PPARγ, ER stress, TRP channels	↑ Programmed cell death	Elimination of malignant cells	(Guzmán, 2003; Velasco et al., 2012; Velasco et al., 2016; Ladin et al., 2016; Dariš et al., 2019; Tomko et al., 2020; Mangal et al., 2024; Ramer et al., 2013; Romano et al., 2013; Vara et al., 2013; Lukhele and Motadi, 2016; Morelli et al., 2014)
Autophagy	ER stress, ceramide-related signaling, autophagy pathways	↑ Autophagic signaling and autophagosome formation	Context-dependent tumor-cell death	(Velasco et al., 2012; Velasco et al., 2016; Ladin et al., 2016; Dariš et al., 2019; Tomko et al., 2020; Mangal et al., 2024)
Inflammatory signaling	NF-κB and related inflammatory pathways	↓ Proinflammatory and prosurvival signaling	Reduced tumor-promoting inflammation	(Guzmán, 2003; Velasco et al., 2012; Velasco et al., 2016; Ladin et al., 2016; Dariš et al., 2019; Tomko et al., 2020)
Angiogenesis	VEGF and endothelial signaling	↓ Endothelial proliferation and vascular support	Reduced tumor angiogenesis	(Guzmán, 2003; Velasco et al., 2012; Velasco et al., 2016; Ladin et al., 2016; Dariš et al., 2019; Tomko et al., 2020)
Invasion and metastasis	EMT-related pathways, ICAM-1, TIMP-1, Id-1, EGFR signaling	↓ Migration, invasion, and metastatic behavior	Reduced metastatic potential	(McAllister et al., 2007; McAllister et al., 2011; Elbaz et al., 2015; Preet et al., 2011; Ramer et al., 2012)
Tumor microenvironment modulation	CB1/CB2, EGFR, stromal and inflammatory signaling	Altered tumor–stroma interactions and local signaling	Potential suppression of tumor progression	(Velasco et al., 2016; Ladin et al., 2016; Dariš et al., 2019; Tomko et al., 2020; Elbaz et al., 2015; Preet et al., 2011)
Immune modulation	CB2 and other cannabinoid-sensitive immune pathways	Modulation of immune-cell activity and cytokine signaling	Context-dependent antitumor or immunosuppressive effects	(Guzmán, 2003; Velasco et al., 2012; Velasco et al., 2016; Ladin et al., 2016; Dariš et al., 2019; Tomko et al., 2020; Taha et al., 2019; Bar-Sela et al., 2020; Waissengrin et al., 2023; Behling-Hess et al., 2025)
Sensitization to anticancer therapy	TRPV2 and other stress-response pathways	Increased susceptibility to temozolomide, bortezomib, imatinib, and other therapies in preclinical models	Potential treatment synergy	(López-Valero et al., 2018; Morelli et al., 2014; Nabissi et al., 2013; Maggi et al., 2022)
Overall biological effect	Multiple interconnected pathways	Reduced viability, proliferation, angiogenesis, and invasion; altered tumor microenvironment	Potential suppression of tumor growth and metastasis, predominantly demonstrated in preclinical models	(Guzmán, 2003; Velasco et al., 2012; Velasco et al., 2016; Ladin et al., 2016; Dariš et al., 2019; Tomko et al., 2020; Guggisberg et al., 2022; Rocha et al., 2014)

Abbreviations. akt, protein kinase B; CB1, cannabinoid receptor 1; CB2, cannabinoid receptor 2; CDK, cyclin-dependent kinase; EGFR, epidermal growth factor receptor; EMT, epithelial–mesenchymal transition; ER, endoplasmic reticulum; ERK, extracellular signal-regulated kinase; GPR55, G protein-coupled receptor 55; ICAM-1, intercellular adhesion molecule 1; MAPK, mitogen-activated protein kinase; mTOR, mechanistic target of rapamycin; NF-κB, nuclear factor kappa B; PI3K, phosphoinositide 3-kinase; PPARγ, peroxisome proliferator-activated receptor gamma; TIMP-1, tissue inhibitor of metalloproteinases 1; TRP, transient receptor potential; TRPV2, transient receptor potential vanilloid 2; VEGF, vascular endothelial growth factor.

Proposed antitumor effects of cannabinoids are supported predominantly by preclinical studies. Clinical evidence demonstrating direct anticancer efficacy remains limited; cannabinoid-mediated immune effects may be bidirectional and context dependent, particularly during immune checkpoint inhibitor therapy.

### 6.2 Antitumor activity across cancer types

Cannabinoids have demonstrated antitumor activity across a broad range of preclinical cancer models; however, translation into clinical benefit remains uncertain, with human evidence largely confined to small early-phase studies and observational data in selected malignancies (Table 5). For most tumor types discussed below, evidence remains preclinical, and no clinical antitumor efficacy has been established.

#### 6.2.1 Glioblastoma

Glioblastoma is the malignancy in which cannabinoid-based therapy has advanced furthest clinically. CB1 and CB2 receptors are expressed in human gliomas, and several studies have reported increased CB2 expression with higher tumor grade. However, receptor expression is heterogeneous and does not independently predict response to cannabinoid therapy (Guzmán, 2003; Velasco et al., 2012; Velasco et al., 2016; Rocha et al., 2014).

Preclinical studies have demonstrated that THC, CBD, synthetic cannabinoid receptor agonists, and THC:CBD combinations inhibit glioma growth through multiple mechanisms, including ceramide accumulation, endoplasmic-reticulum stress, autophagy, apoptosis, oxidative stress, inhibition of angiogenesis, and suppression of migration and invasion (Guzmán, 2003; Velasco et al., 2012; Velasco et al., 2016; Ladin et al., 2016; Rocha et al., 2014). CBD has also demonstrated activity against glioma-initiating and stem-like cells, and activation of transient receptor potential vanilloid 2 (TRPV2) has been proposed to enhance intracellular drug uptake and increase sensitivity to cytotoxic therapy (López-Valero et al., 2018; Rocha et al., 2014).

Among solid tumors, glioblastoma provides the strongest preclinical rationale for combination therapy. In glioma-initiating-cell and xenograft models, THC alone or combined with CBD enhanced the antitumor activity of temozolomide, although efficacy varied according to cannabinoid ratio, dose, and experimental model (López-Valero et al., 2018). Additional studies have suggested radiosensitizing effects, but these observations remain confined to preclinical models (Velasco et al., 2016; Ladin et al., 2016; Rocha et al., 2014).

Clinical evidence remains preliminary. In a pilot study of nine patients with recurrent glioblastoma, intratumoral THC administration was feasible and did not produce major cannabinoid-related toxicity; however, the uncontrolled design and small sample size precluded conclusions regarding efficacy (Guzmán et al., 2006). A subsequent randomized phase Ib trial evaluated nabiximols combined with dose-intense temozolomide in 21 patients with recurrent glioblastoma. Although six-month progression-free survival was identical in the nabiximols and placebo groups (33%), one-year survival was higher in the nabiximols group (83% vs. 44%). Because of the limited sample size and imbalance in early deaths, these findings should be regarded as hypothesis-generating rather than confirmatory (Twelves et al., 2021).

A multicenter randomized phase II trial (ARISTOCRAT) is currently evaluating nabiximols combined with temozolomide in patients with recurrent MGMT-promoter-methylated glioblastoma (Bhaskaran et al., 2024). Nevertheless, no cannabinoid formulation has yet demonstrated a clinically meaningful improvement in progression-free survival or overall survival in adequately powered randomized trials. Cannabinoids should therefore remain investigational and should not replace evidence-based glioblastoma therapy outside prospective clinical studies.

#### 6.2.2 Breast cancer

Breast cancer is among the most extensively investigated malignancies in cannabinoid research. CB1 and CB2 receptors and other components of the endocannabinoid system are expressed across breast-cancer subtypes, although expression varies according to hormone-receptor and HER2 status. CB2 expression is particularly common in HER2-positive tumors, whereas cannabinoid signaling in estrogen receptor-positive and triple-negative breast cancer appears more heterogeneous (Caffarel et al., 2010; McAllister et al., 2007).

Preclinical studies have demonstrated that cannabinoids inhibit breast cancer cell proliferation, angiogenesis, migration, invasion, epithelial–mesenchymal transition, and metastatic dissemination (Ladin et al., 2016; Dariš et al., 2019; Tomko et al., 2020; Caffarel et al., 2010; McAllister et al., 2007; McAllister et al., 2011; Elbaz et al., 2015). Among phytocannabinoids, CBD has shown the most consistent activity, particularly in aggressive and triple-negative breast-cancer models, where inhibition of Id-1 expression, attenuation of EGFR/Akt and PI3K/Akt/mTOR signaling, and induction of oxidative stress and apoptosis have been implicated (McAllister et al., 2007; McAllister et al., 2011; Elbaz et al., 2015). In contrast, although THC and cannabinoid receptor agonists have demonstrated antiproliferative activity in selected HER2-driven models, other studies suggest that cannabinoid signaling may promote tumor progression when immunosuppressive effects predominate, emphasizing the context-dependent nature of cannabinoid biology (Velasco et al., 2012; Caffarel et al., 2010).

In breast cancer, the potential CBD–tamoxifen interaction is particularly relevant, as reduced endoxifen exposure may theoretically compromise tamoxifen efficacy (Parihar et al., 2022). Although the clinical significance remains uncertain, pharmacological-dose CBD should therefore be used cautiously in patients receiving tamoxifen.

Despite extensive preclinical investigation, no prospective clinical trial has demonstrated improvements in response, recurrence-free survival, progression-free survival, or overall survival with cannabinoid therapy in any breast-cancer subtype. Cannabinoids should therefore remain investigational and should not be incorporated into standard breast-cancer treatment outside clinical trials (Braun et al., 2024; Guggisberg et al., 2022; Caffarel et al., 2010).

#### 6.2.3 Prostate cancer

The endocannabinoid system is dysregulated in prostate cancer, and CB1, CB2, transient receptor potential vanilloid 1 (TRPV1), and cannabinoid-metabolizing enzymes are expressed in malignant prostate tissue and prostate-cancer cell lines (Ramos and Bianco, 2012; Czifra et al., 2009). Increased CB1 expression has been associated with higher tumor grade, adverse pathological features, biochemical recurrence, and poorer outcomes in several studies, although its prognostic significance has not been validated for routine clinical use (Ramos and Bianco, 2012; Czifra et al., 2009).

Preclinical studies have demonstrated that cannabinoids inhibit prostate cancer cell proliferation, induce apoptosis and cell-cycle arrest, suppress androgen-receptor signaling, and reduce migration and invasion in both androgen-sensitive and castration-resistant models (Ramos and Bianco, 2012; Czifra et al., 2009).

Clinical evidence remains limited to a phase I dose-escalation and expansion study of pharmaceutical-grade CBD in patients with biochemically recurrent prostate cancer. Epidiolex® was well tolerated at doses up to 800 mg daily but did not demonstrate consistent reductions in prostate-specific antigen levels or improvements in metastasis-free, progression-free, or overall survival (Myint et al., 2023).

Accordingly, cannabinoids currently have no established role as disease-modifying therapy for localized, biochemically recurrent, metastatic hormone-sensitive, or castration-resistant prostate cancer. Future clinical trials should incorporate molecular stratification, pharmacokinetic assessment, and predefined clinical endpoints to better define their therapeutic potential.

#### 6.2.4 Pancreatic cancer

Pancreatic ductal adenocarcinoma (PDAC) is among the most intensively investigated gastrointestinal malignancies in cannabinoid research because of its aggressive biology, therapeutic resistance, and poor prognosis. Components of the endocannabinoid system, including CB1, CB2, GPR55, and cannabinoid-metabolizing enzymes, are expressed in pancreatic-cancer cells and may contribute to tumor growth and survival (Piñeiro et al., 2011; Sharafi et al., 2019; Ferro et al., 2018; Mangal et al., 2024).

Preclinical studies have demonstrated that cannabinoids inhibit pancreatic-cancer growth through multiple mechanisms, including apoptosis, autophagy, oxidative stress, endoplasmic-reticulum stress, and suppression of proliferative signaling (Velasco et al., 2016; Ladin et al., 2016; Dariš et al., 2019; Tomko et al., 2020; Sharafi et al., 2019; Ferro et al., 2018; Mangal et al., 2024). GPR55 has attracted particular interest because it appears to function as a pro-oncogenic receptor in PDAC. Experimental inhibition of GPR55 suppresses MAPK signaling, reduces tumor-cell proliferation, and inhibits tumor growth in preclinical models (Piñeiro et al., 2011; Ferro et al., 2018).

The strongest preclinical evidence comes from a genetically engineered KPC mouse model, in which CBD-mediated inhibition of GPR55 enhanced the antitumor activity of gemcitabine and prolonged survival compared with chemotherapy alone (Ferro et al., 2018). Additional studies have implicated ceramide accumulation, mitochondrial dysfunction, and activation of intrinsic apoptotic pathways in CBD-mediated pancreatic-cancer-cell death (Mangal et al., 2024). However, these findings remain confined to preclinical models.

Clinical evidence is extremely limited. No prospective clinical trial has demonstrated improvements in objective response, progression-free survival, or overall survival with cannabinoid therapy in PDAC (Braun et al., 2024; Guggisberg et al., 2022; Sharafi et al., 2019). Consequently, cannabinoids should remain investigational, although pancreatic cancer represents one of the strongest translational settings for future clinical investigation because of the central role of GPR55 signaling and the potential for combination therapy with gemcitabine.

#### 6.2.5 Lung cancer

Non-small cell lung cancer (NSCLC) has been extensively investigated in cannabinoid research, with CB1 and CB2 receptor expression and cannabinoid signaling demonstrating biological relevance in experimental models (Skórzewska and Gęca, 2024; Guzmán, 2003; Velasco et al., 2012; Velasco et al., 2016; Ladin et al., 2016; Dariš et al., 2019; Tomko et al., 2020; Preet et al., 2011; Ramer et al., 2013; Ramer et al., 2012).

Preclinical studies have shown that THC, CBD, and cannabinoid receptor agonists inhibit NSCLC-cell proliferation, induce apoptosis, and suppress migration, invasion, angiogenesis, and metastatic dissemination (Velasco et al., 2016; Ladin et al., 2016; Dariš et al., 2019; Tomko et al., 2020; Preet et al., 2011; Ramer et al., 2013; Ramer et al., 2012). Proposed mechanisms include COX-2/PPARγ signaling and upregulation of intercellular adhesion molecule-1 (ICAM-1) and tissue inhibitor of metalloproteinases-1 (TIMP-1), thereby reducing tumor invasion and metastatic potential (Ramer et al., 2013; Ramer et al., 2012).

Clinical evidence remains extremely limited. Reports of tumor regression following CBD-containing preparations are confined to isolated case reports and cannot establish therapeutic efficacy (Guggisberg et al., 2022). Observational studies evaluating cannabis use during immune checkpoint inhibitor therapy have yielded conflicting results, with early retrospective studies suggesting inferior outcomes but subsequent prospective studies and systematic reviews failing to confirm a consistent adverse effect (Taha et al., 2019; Bar-Sela et al., 2020; Waissengrin et al., 2023; Behling-Hess et al., 2025).

Accordingly, cannabinoids cannot currently be recommended as adjunctive anticancer therapy for NSCLC, and the effect of concomitant cannabinoid use during immune checkpoint inhibitor therapy remains uncertain.

#### 6.2.6 Colorectal cancer

Alterations in the endocannabinoid system are common during colorectal carcinogenesis. CB1 expression is frequently reduced, whereas GPR55 appears to promote tumor growth, highlighting the complex and sometimes opposing roles of cannabinoid signaling in colorectal cancer (Guzmán, 2003; Velasco et al., 2012; Velasco et al., 2016; Ladin et al., 2016; Dariš et al., 2019; Tomko et al., 2020; Borrelli et al., 2014; Romano et al., 2013; Wang et al., 2008; Hasenoehrl et al., 2018).

Preclinical studies have demonstrated that THC, CBD, cannabigerol (CBG), and cannabichromene (CBC) inhibit colorectal cancer cell growth through apoptosis, oxidative stress, cell-cycle arrest, and suppression of proliferative signaling (Velasco et al., 2016; Ladin et al., 2016; Dariš et al., 2019; Tomko et al., 2020; Borrelli et al., 2014; Romano et al., 2013; Wang et al., 2008; Hasenoehrl et al., 2018). Among the minor phytocannabinoids, CBG reduced aberrant crypt formation and tumor burden in experimental models, whereas CBC inhibited colorectal-cancer-cell growth through reactive oxygen species generation and transient receptor potential ankyrin-1 (TRPA1)-associated signaling (Borrelli et al., 2014; Romano et al., 2013).

Despite encouraging laboratory findings, clinical evidence remains absent. No prospective clinical trial has demonstrated improvements in objective response, progression-free survival, or overall survival with cannabinoid therapy in colorectal cancer (Braun et al., 2024; Guggisberg et al., 2022). Cannabinoids should therefore remain investigational until these promising preclinical observations are validated in well-designed translational and clinical studies.

#### 6.2.7 Hepatocellular carcinoma

The endocannabinoid system is dysregulated in hepatocellular carcinoma (HCC), and CB1, CB2, and related signaling pathways have been implicated in liver-cancer biology (Skórzewska and Gęca, 2024; Guzmán, 2003; Velasco et al., 2012; Velasco et al., 2016; Ladin et al., 2016; Dariš et al., 2019; Tomko et al., 2020; Vara et al., 2013; Afrin et al., 2020). Experimental studies have demonstrated that cannabinoids inhibit hepatocellular carcinoma cell proliferation and promote apoptosis and autophagy through modulation of PPARγ, ERK, Akt, and related signaling pathways (Velasco et al., 2016; Ladin et al., 2016; Dariš et al., 2019; Tomko et al., 2020; Vara et al., 2013; Afrin et al., 2020). PPARγ appears to play a particularly important role, whereas additional studies have implicated CB2 signaling, oxidative stress, and autophagy (Vara et al., 2013; Afrin et al., 2020).

Clinical evidence remains absent. No prospective clinical trial has demonstrated improvements in tumor response, progression-free survival, or overall survival with cannabinoid therapy in HCC. Cannabinoids should therefore remain investigational and should not be considered disease-modifying therapy outside well-designed clinical trials (Braun et al., 2024; Guggisberg et al., 2022).

#### 6.2.8 Head and neck squamous cell carcinoma

Components of the endocannabinoid system are expressed in head and neck squamous cell carcinoma (HNSCC), and selected cannabis extracts and purified cannabinoids have demonstrated antiproliferative activity in experimental models (Skórzewska and Gęca, 2024; Velasco et al., 2016; Ladin et al., 2016; Dariš et al., 2019; Tomko et al., 2020; Nallathambi et al., 2023; Liu et al., 2020). However, cannabinoid activity varies according to tumor subtype, cannabinoid composition, receptor expression, and viral status. Although some cannabis extracts demonstrated greater activity than isolated cannabinoids, these findings remain preclinical and do not establish a clinically meaningful entourage effect (Nallathambi et al., 2023).

An important exception involves HPV-associated HNSCC. Experimental studies have demonstrated that THC and cannabinoid-receptor agonism may promote progression of HPV-positive tumors through activation of p38 MAPK signaling, illustrating the context-dependent and potentially tumor-promoting effects of cannabinoid signaling and raising potential clinical concern given the increasing prevalence of HPV-associated HNSCC (Liu et al., 2020).

Clinical evidence remains limited. No prospective study has demonstrated improvements in tumor response, locoregional control, progression-free survival, or overall survival with cannabinoid therapy in HNSCC. Cannabinoids should therefore remain investigational outside prospective clinical trials (Braun et al., 2024; Guggisberg et al., 2022).

#### 6.2.9 Gynecologic malignancies

Cannabinoid activity has been investigated in several gynecologic malignancies, although available evidence remains almost entirely preclinical. No prospective clinical study has demonstrated disease-modifying activity for any gynecologic cancer (Braun et al., 2024; Guggisberg et al., 2022; Cao et al., 2024; Koltai and Shalev, 2022; Peleg et al., 2024; Marinelli et al., 2020; Lukhele and Motadi, 2016).

##### 6.2.9.1 Ovarian cancer

CBD has demonstrated antiproliferative activity in experimental ovarian-cancer models and may enhance the cytotoxic effects of selected chemotherapeutic agents. Additional studies have reported inhibition of epithelial ovarian-cancer growth through modulation of EGFR/Akt signaling and the intestinal microbiome (Cao et al., 2024; Koltai and Shalev, 2022). However, these findings remain preclinical, and an observational study found no improvement in progression-free or overall survival among patients receiving medical cannabis during PARP-inhibitor maintenance therapy (Peleg et al., 2024).

##### 6.2.9.2 Endometrial cancer

Cannabinoid activity in endometrial cancer appears heterogeneous. Experimental studies have demonstrated that CBD reduces endometrial-cancer-cell viability through TRPV2-associated signaling, accompanied by reactive oxygen species generation, autophagy, and apoptosis, although growth inhibition generally requires relatively high cannabinoid concentrations (Marinelli et al., 2020). No clinical evidence currently supports cannabinoid therapy.

##### 6.2.9.3 Cervical cancer

Only limited laboratory data are available in cervical cancer. CBD has demonstrated concentration-dependent inhibition of tumor-cell proliferation and induction of apoptosis *in vitro*, whereas purified CBD appeared more active than *Cannabis sativa* extracts (Lukhele and Motadi, 2016). No prospective clinical study has demonstrated clinical benefit.

Overall, cannabinoids should remain investigational and should not be considered disease-modifying therapy for gynecologic malignancies outside prospective clinical trials (Braun et al., 2024; Guggisberg et al., 2022).

#### 6.2.10 Hematologic malignancies

Components of the endocannabinoid system are expressed in leukemia, lymphoma, and multiple myeloma, and cannabinoids have demonstrated antiproliferative and proapoptotic activity in experimental models (Irrera et al., 2021; Morelli et al., 2014; Maggi et al., 2022). Experimental evidence suggests that cannabinoids induce apoptosis, inhibit proliferation, and modulate oxidative stress–related pathways in hematologic malignancies, although most data derive from cell-line and animal models (Irrera et al., 2021).

In multiple myeloma, CBD has demonstrated synergistic activity with bortezomib through activation of transient receptor potential vanilloid 2 (TRPV2), enhancing antimyeloma cytotoxicity in preclinical studies (Morelli et al., 2014). Similar TRPV2-dependent mechanisms have been reported in chronic myeloid leukemia, where CBD enhanced the activity of imatinib in experimental models (Maggi et al., 2022). These findings provide a biological rationale for cannabinoid-based combination strategies but remain confined to preclinical investigations.

Clinical evidence remains extremely limited. No prospective clinical study has demonstrated improvements in hematologic response, progression-free survival, or overall survival with cannabinoid therapy in leukemia, lymphoma, or multiple myeloma. Cannabinoids should therefore not be considered substitutes for established hematologic cancer therapy, and future evaluation should be limited to well-designed clinical trials (Braun et al., 2024; Guggisberg et al., 2022; Irrera et al., 2021).

## 7 Translational challenges

Despite extensive laboratory investigation, translation of cannabinoids into effective anticancer therapies has remained limited. Experimental studies consistently demonstrate inhibition of tumor-cell proliferation, induction of apoptosis and autophagy, suppression of angiogenesis, epithelial–mesenchymal transition, and modulation of the tumor microenvironment through CB1, CB2, GPR55, TRP channels, PPARγ, and multiple downstream signaling pathways (Guzmán, 2003; Velasco et al., 2012; Velasco et al., 2016; Ladin et al., 2016; Dariš et al., 2019; Tomko et al., 2020). However, these biological effects have not translated into reproducible improvements in clinical outcomes (Braun et al., 2024; Guggisberg et al., 2022).

Several factors likely contribute to this discrepancy. Most evidence derives from cancer-cell lines and animal models that incompletely recapitulate the biological complexity of human malignancies, including tumor heterogeneity, immune interactions, pharmacokinetic variability, comorbidities, and polypharmacy (Guzmán, 2003; Velasco et al., 2012; Velasco et al., 2016; Ladin et al., 2016; Dariš et al., 2019; Tomko et al., 2020; Guggisberg et al., 2022). In addition, many *in vitro* studies employ cannabinoid concentrations exceeding those achievable with clinically tolerated dosing, while variable oral bioavailability, extensive first-pass metabolism, and uncertain intratumoral drug exposure further limit extrapolation to clinical practice (Huestis, 2007; Grotenhermen, 2003; Lucas et al., 2018; Guggisberg et al., 2022).

Cannabinoid signaling is highly context dependent. Biological responses vary according to tumor type, molecular subtype, receptor expression, immune competence, cannabinoid composition, dose, route and duration of administration, and characteristics of the tumor microenvironment. Under some experimental conditions cannabinoids inhibit tumor growth, whereas under others they may have neutral or even tumor-promoting effects (Guzmán, 2003; Velasco et al., 2012; Velasco et al., 2016; Ladin et al., 2016; Dariš et al., 2019; Tomko et al., 2020; Piñeiro et al., 2011). Moreover, botanical cannabis, purified phytocannabinoids, synthetic cannabinoid receptor agonists, and whole-plant extracts possess distinct pharmacological properties and should not be regarded as interchangeable (Skórzewska and Gęca, 2024; Whiting et al., 2015; Braun et al., 2024; Rock and Parker, 2021; U.S. Food and Drug Administration, 2024b; Therapeutics Limited, 2026).

Clinical development has also been hindered by the absence of validated predictive biomarkers and standardized pharmaceutical formulations. Although altered expression of CB1, CB2, GPR55, FAAH, MAGL, and TRP channels has been reported in multiple malignancies, receptor expression alone does not reliably predict therapeutic response (Guzmán, 2003; Velasco et al., 2012; Pertwee et al., 2010; Piomelli, 2003; Di Marzo et al., 2015; Lu and Mackie, 2016; Maccarrone et al., 2015; Millar et al., 2019; Long and Cravatt, 2011). Most clinical studies have been small, heterogeneous, and designed primarily to evaluate safety or symptom control rather than antitumor efficacy. Consequently, isolated responses and uncontrolled survival differences should be regarded as hypothesis-generating rather than evidence of clinical benefit (Braun et al., 2024; Guggisberg et al., 2022; Guzmán et al., 2006; Twelves et al., 2021).

Among the malignancies reviewed, glioblastoma has progressed furthest into prospective clinical investigation, whereas pancreatic, breast, prostate, lung, and colorectal cancers have generated substantial preclinical evidence supporting continued translational investigation (Caffarel et al., 2010; McAllister et al., 2007; McAllister et al., 2011; Elbaz et al., 2015; López-Valero et al., 2018; Piñeiro et al., 2011; Rocha et al., 2014; Bhaskaran et al., 2024; Ramos and Bianco, 2012; Czifra et al., 2009; Myint et al., 2023; Sharafi et al., 2019; Ferro et al., 2018; Mangal et al., 2024; Preet et al., 2011; Ramer et al., 2013; Ramer et al., 2012; Borrelli et al., 2014; Romano et al., 2013; Wang et al., 2008; Hasenoehrl et al., 2018). Nevertheless, no cannabinoid-based strategy has demonstrated sufficient efficacy to justify incorporation into routine cancer treatment (Braun et al., 2024; Guggisberg et al., 2022; Guzmán et al., 2006; Twelves et al., 2021).

This review has several limitations. It is a narrative rather than a systematic review, and study selection therefore involved author judgment. Preclinical evidence was assessed qualitatively because of substantial heterogeneity in experimental models, compounds, doses, and endpoints. No formal risk-of-bias assessment or quantitative evidence synthesis was performed.

## 8 Future directions

Future cannabinoid research should shift from demonstrating biological activity to defining clinical utility. Priorities include standardized pharmaceutical formulations, biomarker-guided patient selection, rigorous pharmacokinetic characterization, and optimization of drug-delivery strategies capable of achieving therapeutically relevant intratumoral concentrations (Skórzewska and Gęca, 2024; Whiting et al., 2015; Rock and Parker, 2021; U.S. Food and Drug Administration, 2024b; Therapeutics Limited, 2026).

Combination therapy remains a promising area of investigation. Preclinical studies support further evaluation of cannabinoids with chemotherapy, radiotherapy, targeted therapy, and selected immunotherapies, but these combinations require careful assessment of pharmacokinetics, treatment sequencing, drug interactions, immune effects, and overlapping toxicities before clinical adoption (Braun et al., 2024; Zendulka et al., 2016; Buchtova et al., 2023; López-Valero et al., 2018). Particular attention should also be directed toward interactions with immune checkpoint inhibitors, where current evidence remains observational and conflicting (Taha et al., 2019; Bar-Sela et al., 2020; Waissengrin et al., 2023; Behling-Hess et al., 2025).

Future clinical trials should incorporate molecular stratification, validated biomarkers, standardized cannabinoid formulations, and clinically meaningful oncologic endpoints, including objective response, progression-free survival, overall survival, patient-reported outcomes, and quality-adjusted survival. Equally important, clinicians should routinely inquire about cannabinoid use and provide balanced, evidence-based counseling regarding supportive-care benefits, adverse effects, drug interactions, product variability, and the current absence of proven anticancer efficacy (Pergam et al., 2017; Braun et al., 2018; Braun et al., 2024).

## 9 Conclusion

Cannabinoids have an established but selective role in supportive oncology, particularly for chemotherapy-induced nausea and vomiting refractory to standard antiemetic therapy, whereas benefits for cancer-related pain, appetite, sleep, anxiety, and other symptoms remain modest and should be individualized (Braun et al., 2024; Herrstedt et al., 2024; National Comprehensive Cancer Network, 2026; Fallon et al., 2017; Johnson et al., 2010; Jatoi et al., 2002; Strasser et al., 2006; Duran et al., 2010; Grimison et al., 2020; Grimison et al., 2024; National Cancer Institute, 2026). Their supportive-care role should be clearly distinguished from claims of direct anticancer activity.

Although extensive preclinical research demonstrates that cannabinoids influence multiple hallmarks of cancer, these findings have not translated into convincing improvements in objective response, progression-free survival, or overall survival in adequately powered clinical trials (Guzmán, 2003; Velasco et al., 2012; Velasco et al., 2016; Ladin et al., 2016; Dariš et al., 2019; Tomko et al., 2020; Guggisberg et al., 2022; Guzmán et al., 2006; Twelves et al., 2021). Cannabinoids should therefore not be considered disease-modifying therapy or substitutes for established oncologic treatment outside well-designed clinical trials (Braun et al., 2024).

Whether cannabinoid-based therapies ultimately become components of precision oncology will depend on rigorous translational research, biomarker-guided patient selection, standardized formulations, optimized pharmacokinetic strategies, and adequately powered randomized clinical trials. Until such evidence becomes available, clinicians should provide balanced, evidence-based counseling that distinguishes established supportive-care applications from the currently unproven promise of direct anticancer efficacy.
